# Purified [1-^13^C]α-Ketoisocaproate Spin-Polarized Up to 39% with Parahydrogen Enables Real-Time Metabolism Studies in Cancer Cells

**DOI:** 10.1021/acsmeasuresciau.6c00117

**Published:** 2026-08-17

**Authors:** Leander K. May, Lena Börschmann, Denis Moll, Lisa M. Fries, Gonzalo G. Rodriguez, Charlotte von Petersdorff-Campen, Maria D. Santi, Sergey Korchak, Andrei Leonov, Patrick Giavalisco, Stefan Glöggler

**Affiliations:** † NMR Signal Enhancement, 28282 Max Planck Institute for Multidisciplinary Sciences, Am Fassberg 11, Göttingen 37077, Germany; ‡ Center for Biostructural Imaging of Neurodegeneration of the University Medical Center, Von-Siebold-Str. 3a, Göttingen 37075, Germany; § NMR-Based Structural Biology, Max Planck Institute for Multidisciplinary Sciences, Am Fassberg 11, Göttingen 37077, Germany; ∥ Metabolomics Core Facility, 130368 Max Planck Institute for Biology of Ageing, Joseph Stelzmann Str. 9b, Cologne 50931, Germany; ⊥ Advanced Imaging Research Center, University of Texas Southwestern Medical Center, Dallas, Texas 75390, United States; # Department of Biomedical Engineering, University of Texas Southwestern Medical Center, Dallas, Texas 75390, United States

**Keywords:** molecular probes, BCAT, α-ketoisocaproate, parahydrogen, hyperpolarization

## Abstract

Collecting real-time kinetic information about metabolism and downstream metabolites is an emerging field to study living cells and complex systems. Via hyperpolarization techniques in magnetic resonance (MR), signals of metabolites can be enhanced by orders of magnitude. Currently, the main constraints of hyperpolarization techniques are (1) achievable degree of polarization, (2) speed of polarization procedures, (3) availability of metabolites for biological or clinical utility, and/or (4) removal of undesired components (i.e., catalysts, radicals, solvents, etc.), with mainly [1-^13^C]­pyruvate being investigated due to its high metabolic relevance and promising clinical translation. Parahydrogen-induced polarization (PHIP), as an advancing technique, uses parahydrogen (pH_2_), a spin isomer of hydrogen gas, to enhance signal intensities in a matter of seconds to minutes. Here, we introduce an efficient gas/liquid reaction to synthesize a precursor of [1-^13^C]­α-ketoisocaproate-3,3-*d*_3_ (αKIC), namely vinyl-*d*_3_ [1-^13^C]­α-ketoisocaproic acid-3,3-*d*_2_ (VαKIC). αKIC is one of the primary substrates of branched-chain amino acid aminotransferase (BCAT), which is relevant in tumors and neurodegenerative diseases. Hydrogenation of VαKIC with pH_2_ achieved up to 75% ^13^C spin polarization directly after spin order transfer and up to 39% polarization of αKIC (4.1 mM) in a biocompatible solution. These high levels of polarization enabled us to perform *in vitro* studies in human cancer cells and observe the production of the downstream metabolite leucine in real-time. We thereby expand the toolbox of rapidly generated hyperpolarized metabolites and foresee that our synthesis will be suitable to obtain other relevant precursors feasible for hyperpolarization using PHIP-SAH.

## Graphical abstract

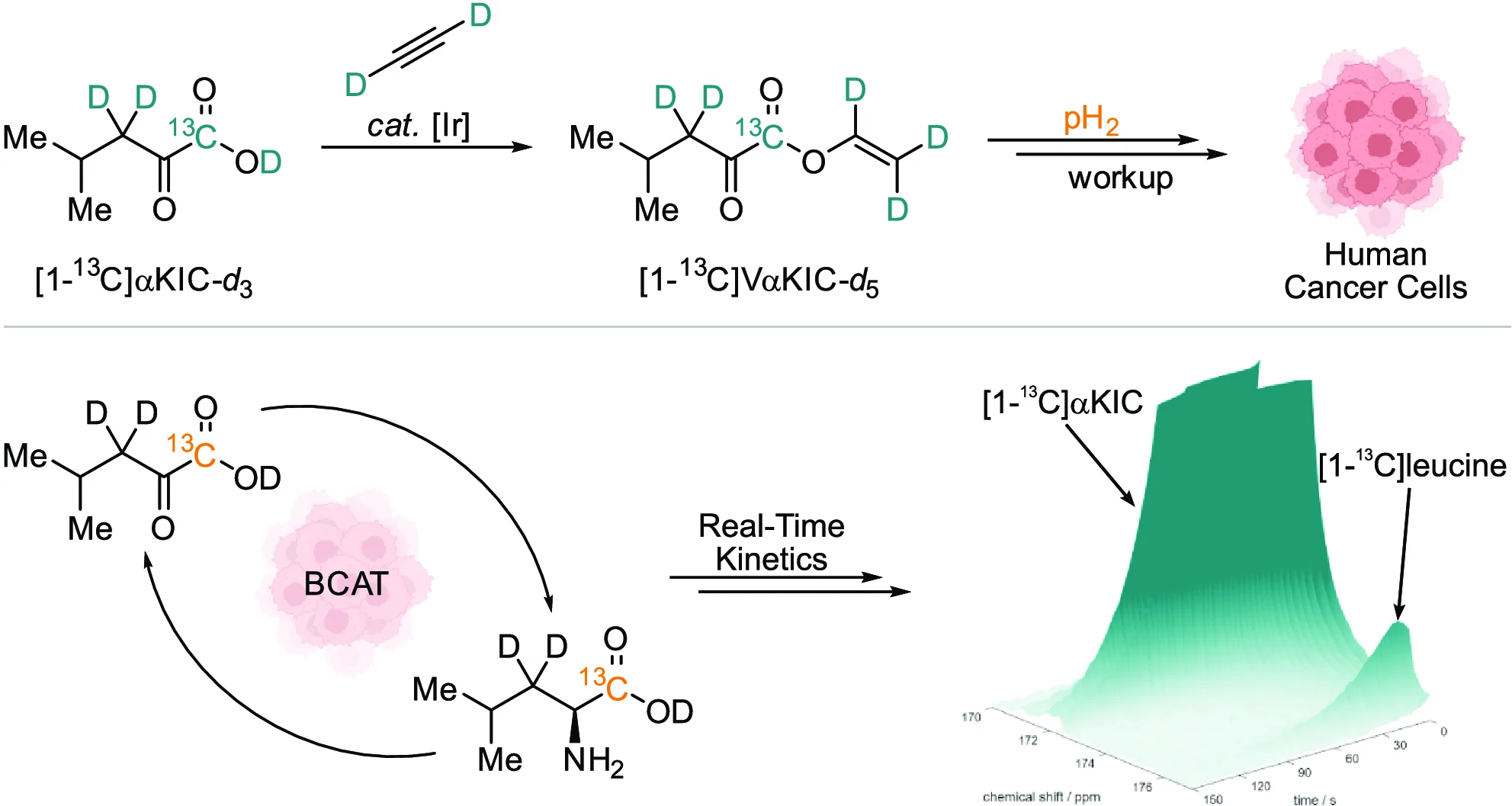

## Introduction

Magnetic resonance (MR) is a versatile tool that offers the possibility of investigating molecular transformations, protein interactions, and cellular metabolism noninvasively via nuclear magnetic resonance (NMR). This helps to understand biological processes and can lead to advancements, e.g., in developing more adequate drugs.^1–5^ For the investigation of larger systems, such as animals or patients, MR enables the investigation of anatomy and metabolism as standard diagnostic tools in the form of magnetic resonance imaging (MRI) and magnetic resonance spectroscopy (MRS), respectively.

Investigating changes in metabolism is of increasing interest, as larger anatomical alterations, such as tumor growth or neurodegeneration, are often linked to an altered metabolism, which already develops in the early stages of these diseases.^6–10^ Visualizing these, especially subtle metabolic alterations, has the potential to contribute to understanding disease developments, reveal new therapeutic pathways, and provide tools for monitoring medication. However, despite its broad application, MR faces limitations due to low sensitivity and spectral congestion, making subtle changes challenging to detect.^11–18^ The reason for this lies in the small difference in the Boltzmann distribution of energetically close spin states, whereby only a small number of spins—on the order of parts per million—contribute to an observable signal.

Hyperpolarized MR tackles these limitations of classical MR by enhancing signal intensities of selected molecules by several orders of magnitude through polarization of nuclear spins. Using this technique, various research applications have been reported, such as production of molecular sensors,^19,20^ reaction monitoring,^21^ and elucidation of mechanistic pathways.^22,23^ In the context of biological processes, hyperpolarization techniques have been employed to enhance signals of key metabolites, such as [1-^13^C]­pyruvate, which can be introduced into an organism and subsequently detected together with its downstream metabolites. Thereby, metabolites become observable and allow for the determination of dysregulation associated with diseases.^11,24–26^

Although the main investigated metabolite using hyperpolarization is pyruvate, it mainly shows metabolism into lactate and is mostly useful to study glycolytic cells. Therefore, expanding the range of available metabolic probes to investigate different aspects of cellular metabolism with hyperpolarization is desirable. One metabolite of interest is α-ketoisocaproate (αKIC), the keto acid of the essential amino acid leucine. Its primary metabolic pathways are through the branched-chain amino acid aminotransferase (BCAT) to leucine and through the branched-chain α-keto acid dehydrogenase complex (BCKD) to CO_2_ and isovaleryl-CoA.^27–29^ The former pathway toward leucine plays an important role in neuronal nitrogen homeostasis and protein synthesis^30–32^ and is being investigated in relation to dysregulation in certain cancers and neurodegenerative diseases, such as Alzheimer’s or Parkinson’s disease.^29,32,33^

For metabolic studies of such dysregulations using hyperpolarized MR, ranging from cells to animals to patients, two aspects are of great importance: (1) a high spin polarization to achieve optimal signal intensity and (2) a fast, scalable approach to obtain hyperpolarized metabolites within minutes. Dissolution Dynamic Nuclear Polarization (dDNP), the most prominent hyperpolarization technique, uses the high spin polarization of electrons at low temperatures (≤4 K)^34–36^ and has already achieved hyperpolarization of αKIC (32 ± 3%, 20 mM) in 2010.^37^ However, αKIC has only been used sparingly for dDNP since then, with studies focusing on animal models and the scalability of this approach.^38–42^

In recent years, parahydrogen (pH_2_)-based methods such as Signal Amplification By Reversible Exchange (SABRE) and Para-Hydrogen-Induced Polarization by Side Arm Hydrogenation (PHIP-SAH) have emerged as alternative methods for scalable production of hyperpolarized molecules. Using pH_2_, the singlet-state spin isomer of hydrogen gas, fast and reliable polarizations of target nuclei such as ^13^C and ^15^N can be achieved at ambient temperature.^12,18,43^

Benefiting from the absence of prior chemical modification of the tracer, SABRE recently reported generation of biocompatible, hyperpolarized αKIC (3.0%, 79 ± 10 mM) in 5–10 min and demonstrated metabolic activity in *Saccharomyces cerevisiae* (Baker’s yeast).^44^ Despite reducing experimental time requirements from dDNP to several minutes, SABRE generally still only yields low polarization values for biocompatible solutions (just very recently up to 10.4% of ∼50 mM αKIC^45^), while specially tailored PHIP-SAH precursors are able to produce hyperpolarized substrates in less than 2 min (∼60–90 s) and yield polarization levels comparable to those of dDNP.^18,19,46,47^ Generating hyperpolarized αKIC via the PHIP-SAH approach therefore combines the advantages of fast hyperpolarization and high levels of signal enhancement. It thus offers an effective alternative, complementing dDNP, for producing hyperpolarized metabolites.

In this work, we introduce a new metabolic precursor, vinyl-*d*_3_ [1-^13^C]­α-ketoisocaproic acid-3,3-*d*_2_ (VαKIC), for the generation of hyperpolarized αKIC. Using targeted deuteration strategies and a new vinylation reaction for the synthesis of the precursor, we achieve about 75% ^13^C polarization directly after hydrogenation and up to 39% in a biocompatible solution (4.1 mM). These high degrees of polarization enabled us to perform initial *in vitro* experiments on human cancer cells to demonstrate enzymatic conversion to hyperpolarized leucine.

## Materials and Methods

### General Remarks

All reactions were carried out in oven-dried glassware (115 °C) that had been cleaned using base (sat. NaOH in propan-2-ol) and acid (HCl in H_2_O) baths, followed by rinsing with deionized water. Unless stated otherwise, air- or moisture-sensitive reactions were carried out in special dried flasks (400 °C) under an inert atmosphere of dry nitrogen using standard Schlenk techniques. Reactions involving gases exceeding ambient pressure were conducted in pressure-safe glassware. All reported reaction temperatures correspond to external bath temperatures. Room temperature was approximately 20 °C.

Special care should be taken when working with gaseous acetylene, and especially when handling pressurized acetylene flasks.

### Materials and Instruments

All chemicals were purchased from commercial suppliers (*SIGMA-Aldrich Chemie GmbH*, *TCI Deutschland GmbH*, *Thermo Fisher Scientific—Life Technologies GmbH*, *Cambridge Isotope Laboratories Inc.*, *VWR International GmbH*) and used as received unless otherwise stated. Solvents for filtrations, transfers, chromatography, and recrystallization were at least of ACS grade. Trimethylsilylacetylene-*d*_1_ was synthesized according to the literature procedure.^48^ Unless stated otherwise, column chromatography was performed using a *puriFlash* *XS 520 Plus* equipped with a UV–vis detector from *Advion Interchim Scientific*. Solvent gradients were automatically calculated using the *InterSoft* *X* software with *Genius* mode from the respective TLC data of the crude compound mixture. As a solid phase, *puriFlash* *SIHP* flash columns (*PF-30SIHP*) in different sizes (*F0012*, *F0020*, *F0040*, *F0080*, or *F0120*), depending on the weight of the crude compound mixture, were used.

Hydrogenation experiments were performed inside the probe head of a *300 MHz Bruker UltraShield Avance III HD* spectrometer, equipped with a *5 mm room temperature PA BBO 300S1 BBF-H-D-05 Z* probe head using *Wilmad-LabGlass High-Throughput* 5 *mm NMR Tubes*. pH_2_, enriched to >98%, was produced using a helium-cooled (20 K) parahydrogen generator (*Sumitomo HC-4A Zephyr Air-Cooled Helium Compressor*, *Sumitomo CH-204 Cold Head*, *Lakeshore Model 335 cryogenic temperature controller* with pH_2_ reaction chamber by *ColdEdge Technologies Inc.*) and delivered via a home-built valve and tubing system.

### Synthesis

#### [1-^13^C]­α-Ketoisocaproic acid-3,3-*d*_3_


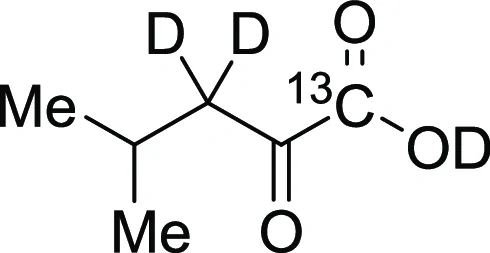


To a 50 mL round-bottom flask, [1-^13^C]­α-ketoisocaproic acid or sodium [1-^13^C]­α-ketoisocaproate (16 mmol, 1.00 equiv) and D_2_O (30 mL) were added. NaOD (40% (w/w) in D_2_O, 20 or 4.8 mmol, 1.25 or 0.30 equiv) was added dropwise, and the solution was stirred for 2 h at room temperature. The reaction progress was monitored by ^1^H NMR to ensure a good degree of deuteration. After completion, the pH of the solution was adjusted to <1.0 using DCl (38% (w/w) in D_2_O), NaCl (∼2 g) was added, and the solution was extracted with Et_2_O (10 × 50 mL). The combined organic fractions were dried over Na_2_SO_4_ and the solvent was carefully removed *in vacuo*. The title compound was obtained as a colorless oil (90%-quant., *D*_β_ = 99%, *D*_γ_ = 20%, Impurity: Et_2_O).

^**1**^ **H NMR** (300 MHz, CDCl_3_): δ = 6.05 (br. s, 0.41 H), 2.76 (ap., 0.02 H), 2.20 (sept., ^3^ *J*_HH_ = 6.5 Hz, 0.80 H, C*H*(CH_3_)_2_), 0.97 (d, ^3^ *J*_HH_ = 6.7 Hz, 6 H, C*H*_3_) ppm.

^**2**^ **H NMR** (46 MHz, CDCl_3_): δ = 6.16 (s, 1.5 D), 2.77 (s, 2 D, C*D*_2_) ppm.

^**13**^ **C­{** ^**1**^ **H,** ^**2**^ **H} NMR** (75 MHz, CDCl_3_): δ = 196.0 (d, ^1^ *J*_CC_ = 59.9 Hz, C_2_), 160.1 (^13^C_1_), 45.5 (d, ^2^ *J*_CC_ = 13.3 Hz, C_3_), 24.5 (C_4_), 22.5 (C_5_) ppm.

**HRMS-ESI­(−)**: calculated *m*/*z* for C_5_^13^C_1_H_7_D_2_O_3_ ([M – D]^−^): 132.0716; found: 132.0715.

#### Vinyl-*d*_3_ [1-^13^C]­α-ketoisocaproic acid-3,3-*d*_2_


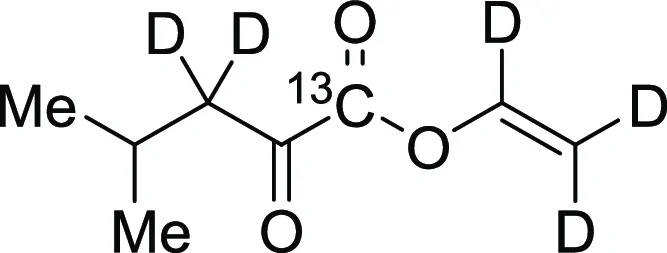


To a 1 L *J Young* cylindrical Schlenk flask under N_2_ atmosphere, [Ir­(COD)­Cl]_2_ (178 μmol, 3 mol %), Na_2_CO_3_ (611 μmol, 10 mol %), and dry DCM (5 mL) were added. [1-^13^C]­α-ketoisocaproic acid-3,3-*d*_3_ (5.96 mmol, 1.00 equiv) in dry DCM (5 mL) was added, and the suspension cooled to −20 °C. The flask was evacuated until the solvent started to boil and then slowly backfilled with freshly generated acetylene-*d*_2_ gas to ∼1.5 bar. The flask was tightly sealed and the suspension vigorously stirred at 48 °C for 5 d. Afterward, the suspension was cooled to 0 °C and the pressure slowly released. The suspension was filtered, the volume reduced to about 2–3 mL, and the residue purified by column chromatography (hexane:EtOAc, 95:5). After removing the solvent *in vacuo* and later a stream of N_2_, the title compound was obtained as a slightly yellow oil (2.68 mmol, 45%, *D*_β_ = 96%, *D*_γ_ = 20%, *D*_vinyl_ = 97–98%).

^**1**^ **H NMR** (300 MHz, acetone-*d*_6_): δ = 7.29 (ap., 0.03 H), 5.08 (ap., 0.02 H), 4.81 (ap., 0.03 H), 2.78 (ap., 0.08 H), 2.15 (sept., ^3^ *J*_HH_ = 6.8 Hz, 0.80 H, C*H*(CH_3_)_2_), 0.96 (d, ^3^ *J*_HH_ = 6.4 Hz, 6 H, C*H*_3_) ppm.

^**2**^ **H NMR** (46 MHz, acetone-*d*_6_): δ = 7.32 (s, 1 D, C*D* = CD_2_), 5.11 (s, 1 D, *E*-CD = C*D*_2_), 4.85 (s, 1 D, *Z*-CD = C*D*_2_), 2.79 (s, 2 D, C*D*_2_) ppm.

^**13**^ **C­{** ^**1**^ **H,** ^**2**^ **H} NMR** (75 MHz, acetone-*d*_6_): δ = 193.1 (d, ^1^ *J*_CC_ = 67.5 Hz, C_2_), 158.6 (^13^C_1_), 141.5 (d, ^2^ *J*_CC_ = 2.6 Hz, C_6_), 100.1 (d, ^3^ *J*_CC_ = 3.7 Hz, C_7_), 47.4 (d, ^2^ *J*_CC_ = 15.3 Hz, C_3_), 24.6 (d, ^3^ *J*_CC_ = 1.2 Hz, C_4_), 22.6 (C_5_) ppm.

**HRMS-ESI­(+)**: calcd *m*/*z* for C_7_^13^C_1_H_7_D_5_O_3_Na ([M + Na]^+^) 185.1026; found 185.1027.

#### Generation of Acetylene-*d*_2_


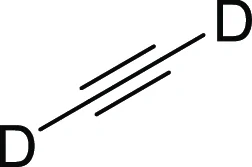


To a 50 mL two-neck round-bottom flask equipped with a rubber septum, D_2_O (7.5 mL), NaOD (40% (w/w) in D_2_O, 2.5 mL, 41.0 mmol, 1.00 equiv), and Bu_4_NCl (200 mg, 720 μmol, 2 mol %) were added. The flask was connected to a home-built valve and tubing system, and vigorous stirring was started. Trimethylsilylacetylene-*d*_1_ was slowly added to the solution to start the acetylene-*d*_2_ generation. The gas was directly transferred to a *J Young* Schlenk flask without analysis or storage.

### General Procedure for PHIP Experiments

VαKIC hyperpolarization samples were prepared from stock solutions of the hydrogenation catalyst, [1,4-bis­(diphenylphosphino)­butane]­(1,5-cyclooctadiene)­rhodium­(I) tetrafluoroborate (12.3 mM, 195 μL for 3.4 mM VαKIC or 14.9 mM, 185 μL for 13 and 68 mM VαKIC, CAS: 79255-71-3, *SIGMA-Aldrich Chemie GmbH*), and VαKIC (∼200 mM, 5 μL for 3.4 mM VαKIC or 15 μL for 13 mM VαKIC or ∼930 mM, 15 μL for 68 mM VαKIC) in acetone-*d*_6_ to yield a final concentration of 12 or 13.8 mM of the catalyst and 3.4, 13, or 68 mM of VαKIC. The freshly prepared hyperpolarization samples were stored at −25 °C until use. Hydrogenation using pH_2_ was performed using a home-built setup inside the spectrometer at 8 bar for 12 or 18 s. Transfer of spin polarization from pH_2_-^1^H to the ^13^C moiety was done using the MINERVA (^3^ *J*_CH_ = 3.12 Hz, ^3^ *J*_HH_ = 7.15 Hz) pulse sequence with an additional 90° pulse at the end to return the magnetization to the *z* direction for the workup procedure.^49^

Similar to what was described previously,^49^ cleavage of the ethyl side arm and purification of the hyperpolarized VαKIC sample were performed on top of the spectrometer in front of a magnetic plate with a strength of approximately 400 mT to minimize signal loss due to *T*_1_ relaxation. For this purpose, aqueous Na_2_CO_3_ (100 mM, 120 μL, D_2_O) was added while the sample was manually lifted out of the spectrometer. This was followed by 2 s of N_2_ bubbling and 0.5 s of settling. The acetone-*d*_6_ was evaporated *in vacuo* in a 65 °C water bath for 7 s. The residual suspension was transferred to a preloaded syringe containing an aqueous HEPES buffer solution (100 mM, 100 μL, D_2_O, pD = 3) and filtered through a syringe filter (*Whatman Puradisc 13 mm GMF*, *pore size: 0.7* *μm*, *Thermo Fisher Scientific—Life Technologies GmbH*) into a new NMR tube to remove residual catalyst and adjust the pD to physiological conditions.

### Cell Cultivation and Preparation for PHIP Experiments

Human Burkitt lymphoma cells (RAMOS and DG-75, DSMZ no.: ACC 603 and ACC 83, respectively) and human T cell leukemia cells (JURKAT, DSMZ no.: ACC 282) were cultured in supplemented DMEM (high d-glucose, l-glutamine, pyruvate, 10% FBS, and 1% penicillin/streptomycin) to around 70% confluence in a humidified incubator (36.8 °C, 5% CO_2_). For the experiments, the cells (20–60 million) were harvested, pelleted by centrifugation (room temperature, 5 min, 3000 rcf), and the supernatant removed. Fresh medium (180 μL) was added, and the cell suspension (160 μL) was transferred to a standard *Wilmad-LabGlass High-Throughput* *5* *mm NMR Tube* directly before measurement. During hyperpolarization of the VαKIC sample, the cell suspension was kept at 37 °C in an *Eppendorf ThermoMixer C* heating block. No precipitation of the cells was observed during the time necessary for hyperpolarization of the sample. Cell viability was measured before and after the PHIP experiments using trypan blue staining.

The purified, hyperpolarized αKIC was administered directly to the already prepared cell suspension, vigorously mixed using a long, nonpointed cannula used for administration of the αKIC, and the NMR tube was transferred back inside the probe head of the spectrometer for acquisition. ^13^C NMR spectra were recorded at 37 °C in a pseudo-2D sequence (64 repetitive 1D-^13^C NMR spectra over approximately 3 min) with a flip angle of 18° and a repetition time of *t*_r_ = 3 s. After the experiment, slight precipitation (approximately 1–2 mm of supernatant) of a cell pellet was observed at the bottom of the NMR tube.

### Preparation of Cell Samples for Mass Spectrometric Analysis

To prove that the formation of leucine stems from the metabolism of the used cancer cells, different control experiments were performed and analyzed via mass spectrometry. For this purpose, four variations of the cell experiments were performed (each *n* = 3) using the JURKAT cell line:

1. normal procedure (cells + medium with 10% FBS + VαKIC)
2. without FBS (cells + medium without FBS + VαKIC)
3. without cells (medium with 10% FBS + VαKIC)
4. without VαKIC (cells + medium with 10% FBS)

Experiments 1–3 were conducted as reported above, and representative summed ^13^C spectra are shown in Figure S4 in the Supporting Information (sum over all 64 scans). For experiment 4, the cell pellet was resuspended in fresh media, subsequently combined with ∼200 μL of an aqueous solution of Na_2_CO_3_ (100 mM, 100 μL, D_2_O) and HEPES buffer solution (100 mM, 100 μL, D_2_O, pD = 3), which were also used for the PHIP experiments, and left to stay at 37 °C for about 5 min. Afterward, the cell suspension was handled similarly to experiments 1–3 (see below).

For the preparation of the cell samples for mass spectrometric analysis, the cell suspension was immediately taken out of the NMR spectrometer after the PHIP experiment and transferred to a 1.5 mL *Eppendorf Safe-Lock* tube. The tube was centrifuged (4 °C, 5 min, 3000 rcf), and the supernatant was transferred to a separate 1.5 mL tube. The residual cell pellet was washed with cold DPBS (200 μL, 4 °C) and centrifuged (4 °C, 5 min, 3000 rcf), the supernatant was removed, and the cell pellet was immediately frozen in liquid nitrogen (−196 °C). All tubes were immediately stored on dry ice (−79 °C) for transportation and sent to the *Metabolomics Core Facility* of the *Max Planck Institute for Biology of Aging* the next day.

### Targeted Liquid Chromatography/Anion-Exchange Chromatography High-Resolution Mass Spectrometry-Based (LC/AEX-HRMS) Analysis

For the mass spectrometric analysis, the dried medium samples and the cell pellet were reconstituted in 700 μL of LCMS-grade acetonitrile:methanol:water (2:2:1 [v:v:v], *Biosolve*), containing 250 nM of U–^13^C^15^N amino acid mix (*Cambridge Isotope Laboratories, Inc.*, *MSK_A2-1.2*) and 20 ng of citric acid-*d*_4_ and succinic acid-*d*_4_ (*SIGMA-Aldrich Chemie GmbH*). The resuspended cell pellet was tip sonicated using 10 pulses of 0.5 s and 65% amplitude (*BANDELIN SONOPLUS mini20*) before incubating them for 30 min at 4 °C using an orbital shaker at 1500 rpm (*Eppendorf ThermoMixer C*). The extracts were centrifuged for 10 min at 21,000 × *g* and 4 °C, and the cleared supernatant was transferred to a fresh 1.5 mL *Eppendorf* tube and dried down using a SpeedVac (*LaboGene, Scan Speed 40*).

The dried samples were resuspended in 1 mL of UPLC water (*Biosolve*) and incubated for 10 min at 1500 rpm using an orbital shaker at 4 °C (*Eppendorf ThermoMixer C*). The resuspended extracts were centrifuged again for 10 min at 21,000 × *g* at 4 °C, and the cleared supernatant was further diluted 1:10 [v:v] in UPLC water (*Biosolve*) and transferred to fresh 1.5 mL *Eppendorf Safe-Lock* tubes.

For the LC-HRMS analysis of amine-containing compounds, it was performed as described previously.^50^

**In brief:** 50 μL of the diluted sample was mixed with 25 μL of 100 mM sodium carbonate (*SIGMA-Aldrich Chemie GmbH*), followed by the addition of 25 μL of 2% [v/v] benzoyl chloride (*SIGMA-Aldrich Chemie GmbH*) in acetonitrile (*Biosolve*). The sample was mixed immediately by pipetting the solvents up and down before transferring it to a glass vial with a 300 μL glass insert (*Chromatographie Zubehör Trott*, *Germany*). The derivatized samples were kept at room temperature (20 °C) until analysis.

For the LC-HRMS analysis, 2 μL of the derivatized sample was separated on an *Acquity* *PREMIER HSS T3 UPLC* column (100 × 2.1 mm, 1.8 μm particle size, *Waters*) using a *Vanquish Horizon UHPLC* system (*Thermo Fisher Scientific—Life Technologies GmbH*). The flow rate was set to 400 μL/min using a binary buffer system consisting of buffer A (10 mM ammonium formate and 0.15% [v/v] formic acid (*Biosolve*) in water) and buffer B (acetonitrile with 0.1% [v/v] formic acid). The column temperature was set to 40 °C, while the LC gradient was: 0% B at 0 min, 0–15% B (0–4.1 min); 15–17% B (4.1–4.5 min); 17–55% B (4.5–11 min); 55–70% B (11–11.5 min); 70–100% B (11.5–13 min); 100% B (13–14 min); 100–0% B (14–14.1 min); 0% B (14.1–19 min). The mass spectrometer (*Q Exactive Plus*, *Thermo Fisher Scientific—Life Technologies GmbH*) was operating in positive ionization mode, recording the *m*/*z* 100–1000 at a resolution of 140,000 in full scan mode. The heated ESI source settings of the mass spectrometer were: spray voltage 3.5 kV, capillary temperature 300 °C, sheath gas flow 60 AU, aux gas flow 20 AU at 330 °C, and the sweep gas was set to 2 AU. The RF lens was set to a value of 60 AU.

The AEX-MS analysis of anionic metabolites was performed as described previously.^51^

**In brief:** 80 μL of diluted sample was transferred to polypropylene autosampler vials (*Chromatographie Zubehör Trott*, *Germany*). For AEX-MS analysis, 5 μL of sample was injected in push full mode with an overfill factor of 1, using a *Dionex Ion Chromatography* system (*Integrion*, *Thermo Fisher Scientific—Life Technologies GmbH*) equipped with a *Dionex IonPac AS11-HC* column (2 mm × 250 mm, 4 μm particle size, *Thermo Fisher Scientific—Life Technologies GmbH*) and a *Dionex IonPac AG11-HC guard* column (2 mm × 50 mm, 4 μm, *Thermo Fisher Scientific—Life Technologies GmbH*). The column temperature was held at 30 °C, while the autosampler was set to 6 °C. A potassium hydroxide gradient was generated using a potassium hydroxide cartridge (*Eluent Generator*, *Thermo Fisher Scientific—Life Technologies GmbH*), which was supplied with LCMS-grade Milli-Q water. The metabolite separation was carried out at a flow rate of 380 μL/min, applying the following gradient conditions: 0–3 min (10 mM KOH); 3–12 min (10–50 mM KOH); 12–19 min (50–100 mM KOH); 19–22 min (100 mM KOH); 22–23 min (100–10 mM KOH). Before the next injection, the column was re-equilibrated at 10 mM KOH for 3 min. The mass spectrometer (*Q Exactive HF*, *Thermo Fisher Scientific—Life Technologies GmbH*) was operating in negative ionization mode, recording *m*/*z* 77–770 in full scan mode at a resolution of 240,000. The heated ESI source settings of the mass spectrometer were: spray voltage 2.75 kV, capillary temperature 275 °C, sheath gas flow 50 AU, aux gas flow 14 AU at 380 °C, while the sweep gas was set to 3 AU. The RF lens was set to a value of 45 AU.

Integration of analyte peaks was performed using the *TraceFinder* software (*Version 5.1*, *Thermo Fisher Scientific—Life Technologies GmbH*). The identity of each compound was validated by an in-house library generated with authentic reference compounds. Compounds were accepted when they matched a mass accuracy of <3 ppm and a retention time (RT) tolerance of <0.05 min. To account for any RT shifts, a comprehensive standard mix was injected before and after each experimental sequence.

If not indicated otherwise, all solvents were of LCMS grade or higher quality and purchased from *Biosolve* or *SIGMA-Aldrich Chemie GmbH*.

## Results and Discussion

As a first step, we designed an efficient, two-step synthetic route to obtain a feasible αKIC precursor for PHIP-SAH, utilizing acetylene gas as the source to introduce the unsaturated moiety (see Figure 1 **A**). Targeted incorporation of deuterium in the β-position of the main framework, αKIC, increases *T*_1_ relaxation times by reducing dipolar couplings to neighboring nuclear spins and increases achievable polarization levels by reducing redistribution effects during the polarization transfer due to scalar couplings. We achieved a high degree of deuteration (*D*_β_ = 99%) by stirring αKIC in a basic D_2_O solution, yielding the desired deuterated product with an excellent yield (90%-quant.). Additional deuteration of the γ-position was observed, with about 20% deuterium incorporation. The unsaturated moiety was introduced as a deuterated vinyl-side arm via an efficient gas/liquid reaction of αKIC-*d*_3_ and acetylene-*d*_2_ gas in the presence of a metal catalyst. After 5 d, full conversion to the product, VαKIC, was achieved without significant side-product formation. The product’s stability and volatility during the purification resulted in a yield of 45%.

**Figure 1:**
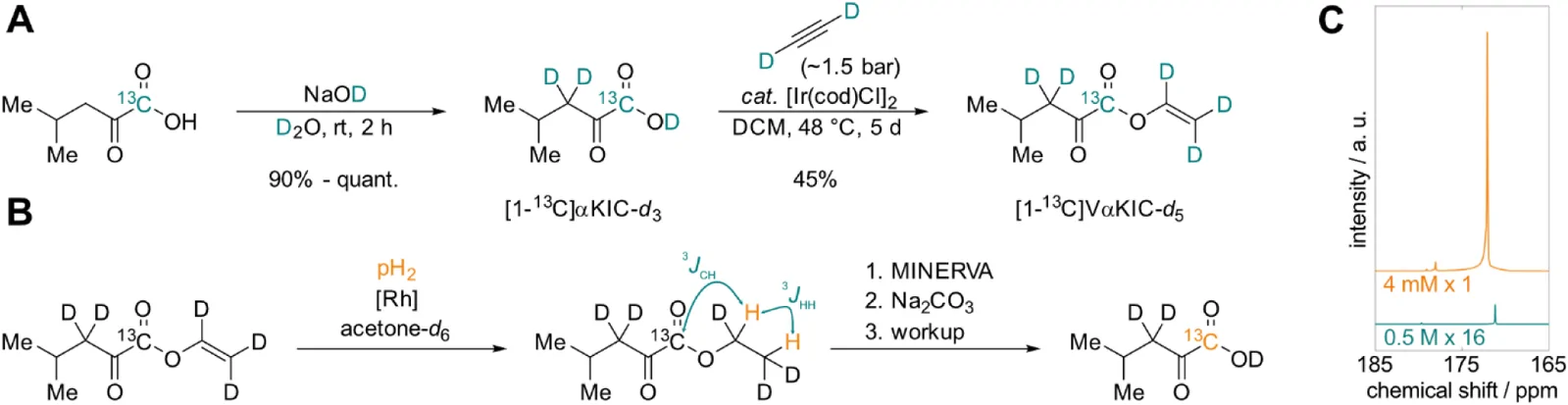
Synthesis of VαKIC, PHIP-SAH procedure for VαKIC to obtain hyperpolarized αKIC, and comparison of hyperpolarized αKIC and thermally polarized [1-^13^C]­pyruvate ^13^C NMR spectra. **A.** VαKIC was synthesized in two steps, starting with αKIC via initial deuteration and subsequent iridium-catalyzed incorporation of the vinyl-side arm using acetylene-*d*_2_. Isotope labels are marked in cyan. **B.** Overview of the hyperpolarization procedure. Hyperpolarized nuclei are marked in orange. **C.** Comparison of hyperpolarized αKIC (orange, ∼4 mM, ∼0.2 mL buffered D_2_O, 310 K, 1 scan) and thermally polarized sodium [1-^13^C]­pyruvate (cyan, 0.5 M, 0.2 mL D_2_O, 298 K, 4 scans, 4 × scale) ^13^C NMR spectra. A small αKIC·hydrate peak (∼178.1 ppm) besides the main αKIC peak (∼172.1 ppm), with an intensity of about 3% of the αKIC peak, can be observed.

Albeit presenting a very efficient reaction for the introduction of vinyl groups, the depicted reaction poses a significant risk when not handled with care. It is, therefore, explicitly advised to stay within the pressures reported in this work.

We propose two possible modes of action based on π-acid catalysis of iridium­(I) or a migratory insertion of the acetylene into an Ir–D bond and subsequent reductive elimination.^52^ In the first mechanism, coordination of the acetylene-*d*_2_ to monomeric iridium­(I) activates the alkyne toward nucleophilic attack by the carboxylate, forming an iridium­(I)-σ-alkenyl intermediate. Deuterodemetalation yields the desired vinylated product and regenerates the active iridium­(I) catalyst (see Figure 2, left). The second mechanism is based on a subsequent oxidative addition of the carboxylic acid via the O–D bond after initial acetylene-*d*_2_ coordination. Migratory insertion of the acetylene-*d*_2_ into the Ir–D bond leads to an iridium­(III)-σ-alkenyl intermediate, which undergoes reductive elimination to form the desired vinylated product and regenerates the active iridium­(I) catalyst (see Figure 2, right). A more detailed discussion of the proposed mechanisms can be found in the Supporting Information (SI).

**Figure 2:**
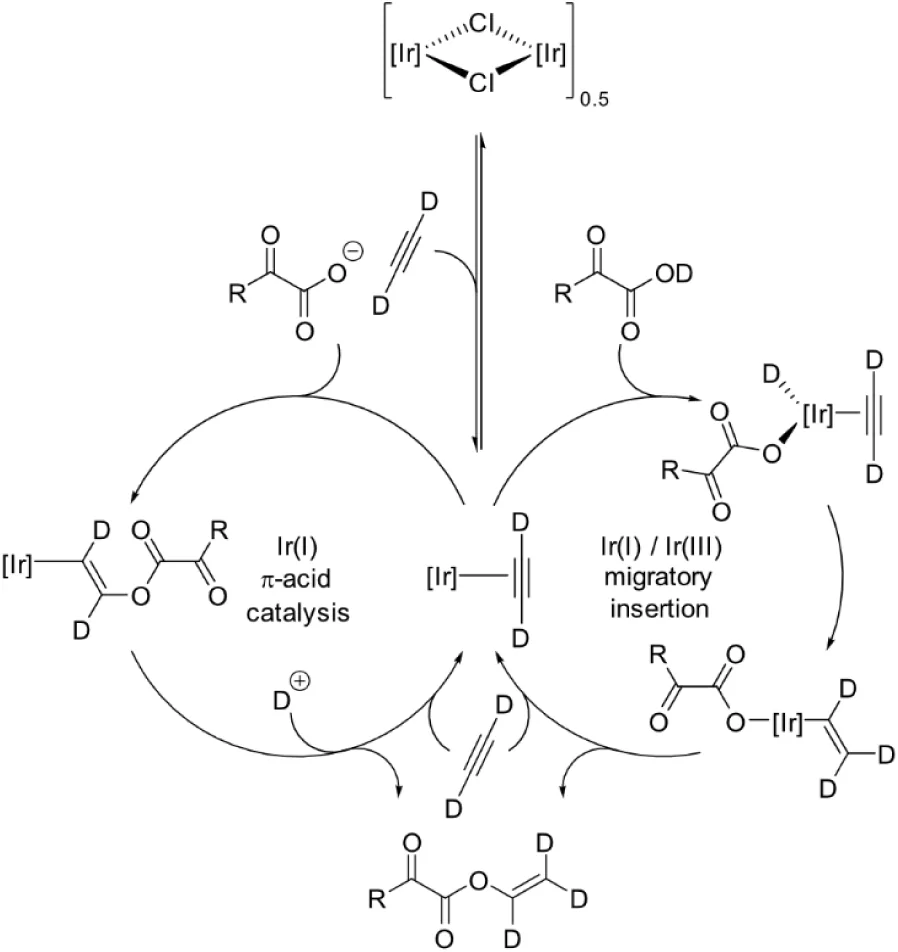
Proposed mechanisms of the vinylation of αKIC. π-acid-catalyzed activation of acetylene-*d*_2_ by monomeric iridium­(I) followed by nucleophilic attack of the carboxylate to form an iridium­(I)-σ-alkenyl intermediate. Deuterodemetalation yields the desired vinyl ester and regenerates the active iridium­(I) catalyst (left). Oxidative addition of the carboxylic acid via the O–D bond and subsequent insertion of the acetylene-*d*_2_ into the Ir–D bond to form an iridium­(III)-σ-alkenyl intermediate. Reductive elimination yields the desired vinyl ester and regenerates the active iridium­(I) catalyst (right).

With VαKIC at hand, we started investigating the hyperpolarization ability under PASADENA (Parahydrogen And Synthesis Allow Dramatically Enhanced Nuclear Alignment) conditions for PHIP-SAH using the MINERVA (Maximizing Insensitive Nuclear Enhancement Reached Via parahydrogen Amplification) pulse sequence to transfer polarization after hydrogenation from the pH_2_-derived hydrogen atoms to the ^13^C nucleus.^49^ The detailed procedure can be found in the Supporting Information (SI) and is based on our previously reported procedures (see Figure 1 **B**).^46,49,53^ In short, initial hydrogenation experiments were performed using a homogeneous rhodium catalyst and 3.4 mM solutions of VαKIC in acetone-*d*_6_ to optimize the reaction temperature, pH_2_ bubbling time, catalyst concentration, and the workup procedure. The latter was performed to obtain an aqueous solution of the free carboxylic acid at a physiological pH (∼7.0–7.5), and to remove the catalyst and the organic solvent.

Performing hydrogenation experiments without the subsequent workup procedure resulted in high ^13^C polarization levels of 75% (∼130,000-fold enhancement at 7 T) on the 1-^13^C of ethyl-1,2,2-*d*_3_ [1-^13^C]­α-ketoisocaproic acid-3,3-*d*_2_ (EtαKIC), the hydrogenated product. After the workup procedure, good polarization levels of about 35% (1.2 mM αKIC, ∼60,000-fold enhancement at 7 T) were maintained, similar to the trends of analogously produced [1-^13^C]­pyruvate.^19,49^ The loss in concentration (∼65%) is a result of the workup procedure and the volatility of both the hydrogenated product, EtαKIC, and αKIC. Assuming a similar *T*_1_ relaxation (see below) during the workup process, the loss of polarization, due to the additional time required for the workup step (approximately 30 s), can be approximated to be 30–40% of the initial polarization level. Strong pH changes and chemical conversion are additional influences contributing to the reduced polarization level. Further modifications involving the use of sodium ascorbate to decrease polarization loss, resulting from the formation of paramagnetic species during the workup,^54,55^ did not lead to an increase in polarization.

Targeting real-time metabolic measurements *in vitro* and *in vivo*, the substrate concentrations were adjusted to 13 mM and 68 mM (Note: typical concentrations for clinical applications are ∼250 mM of hyperpolarized [1-^13^C]­pyruvate^56^), respectively, and the optimized hydrogenation conditions modified accordingly. An upper limit of the catalyst concentration was reached for the higher concentration samples at the solubility maximum (approximately 13.8 mM in acetone-*d*_6_). Additionally, the temperature was adapted to 310 K for cell experiments to ensure the survival of the cells during the kinetic measurement inside the spectrometer. Here, polarization levels of about 72% before workup and 39% (4.1 mM αKIC) after workup were achieved (see Figure 1 **C**). For 68 mM samples, polarization levels of about 27% after workup were achieved while retaining 28% of the initial concentration (19 mM αKIC). Table 1 shows the optimized hyperpolarization conditions, remaining substrate concentration, and achieved polarization level. It is worth mentioning that a decrease in concentration of approximately 5% was observed directly after hydrogenation of the 19 mM samples, highlighting the volatility of EtαKIC at higher concentrations. No such effect was observed at a concentration of 3.4 mM.

**Table 1:** Concentration of the Precursor V_αKIC_ before Hyperpolarization (*c*_VαKIC_), ^13^C Polarization Level (*P*_^13^C_) and Concentration of the Free, Hyperpolarized αKIC after Aqueous Workup (*c*_αKIC_) and Optimized Conditions for Hyperpolarization of Different Sample Concentrations

*c*_VαKIC_/mM	*P*_^13^C_/%	*c*_αKIC_/mM	conditions
3.4 ± 0.2	35 ± 6	1.2 ± 0.3	320 K, 12 s pH_2_, 10 mM [Rh]
13 ± 1	39 ± 3	4.1 ± 0.2	310 K, 18 s pH_2_, 13.8 mM [Rh]
68 ± 3	27 ± 4	19 ± 3	320 K, 18 s pH_2_, 13.8 mM [Rh]^a^

a Optimized conditions were based on 3.4 and 13 mM samples.

The achieved polarization levels of about 27% (19 mM αKIC) should show a reasonably good signal enhancement to target initial *in vivo* experiments, as could be shown again very recently by Petersen et al.^45^ In their work on SABRE-polarized αKIC, they could show decent uptake of αKIC into the healthy mouse brain and metabolic activity to detect hyperpolarized leucine using 50 mM solutions of hyperpolarized αKIC, albeit only having polarization levels of about 11%. Compensating for the lower tracer concentration, our higher polarization levels should yield comparable results without further optimization of our hyperpolarization procedure in respect to the higher concentration (i.e., increase of pH_2_ bubbling time for higher substrate concentrations).

Current limitations for the substrate recovery during the workup lie in the removal of organic solvent *in vacuo* and the subsequent filtration step. Due to the rapid removal of the organic solvent (within 7 s), potential coevaporation of the rather volatile target molecule leads to reduced concentrations of the final aqueous solution. Additional entrapment of the product within the precipitated catalyst residue might lead to an additional reduction in concentration. To optimize the product retention, a milder but fast removal of the organic solvent is necessary. For that purpose, liquid–liquid extractions or slower evaporation at a lower vacuum might present viable alternatives. Both methods proved to be efficient in previous SABRE studies, retaining ∼56% (70 mM starting concentration)^44^ and ∼77% (10 mM starting concentration)^45^ of the substrate, respectively. Additional higher volumes of the final aqueous solution would further reduce losses in the filtration step.

We further investigated the longitudinal *T*_1_ relaxation time of hyperpolarized αKIC to estimate the time scale over which the hyperpolarized metabolic tracer can be observed *in vitro*. Performing the workup in D_2_O instead of H_2_O prolonged *T*_1_ relaxation by 37%, from 54 to 74 s, enabling monitoring over several minutes (see Figures 3 **A** and S1–S3 in the Supporting Information).

**Figure 3:**
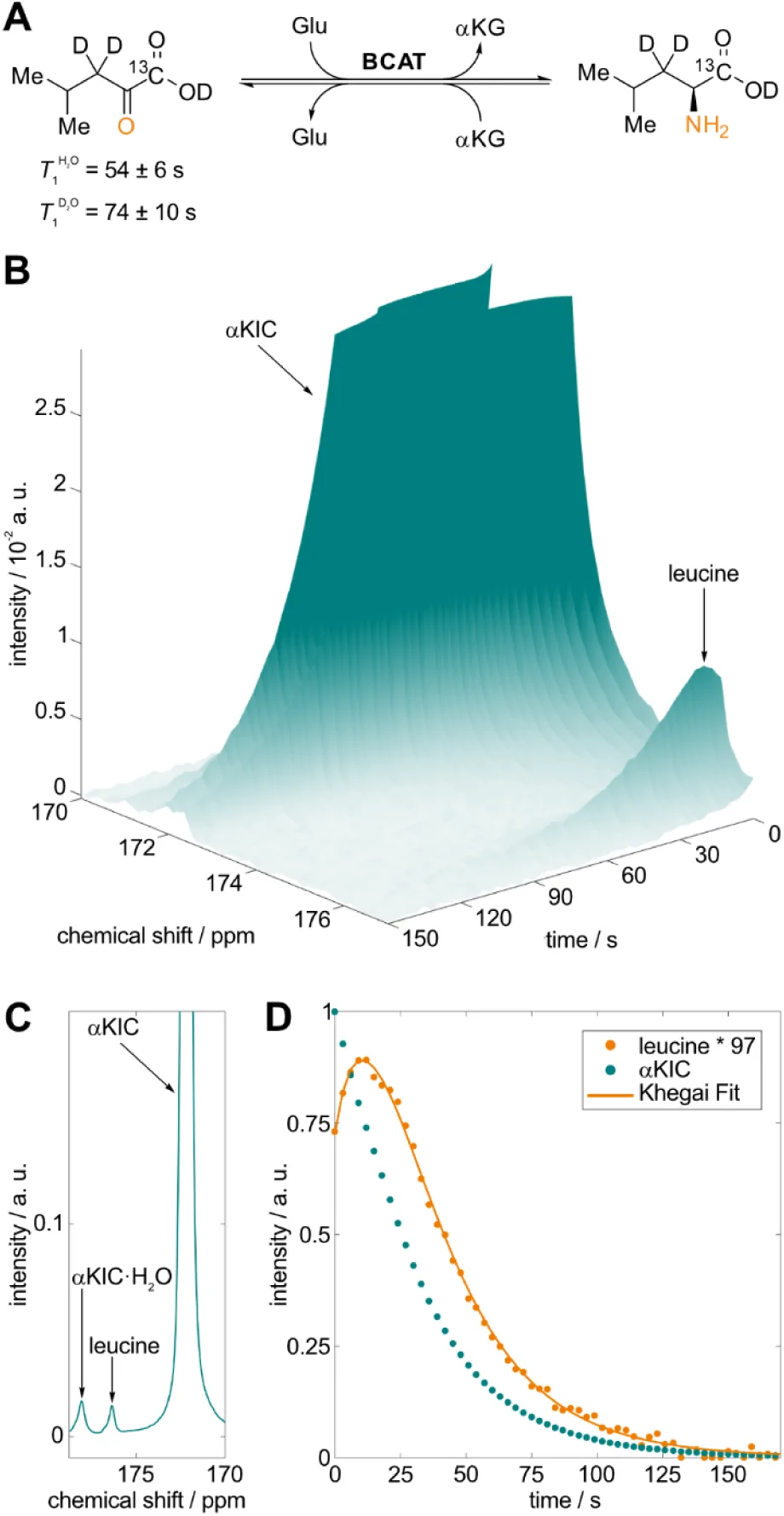
Scheme of probed metabolism of αKIC, excerpt of ^13^C NMR spectra of JURKAT cells after injection of hyperpolarized αKIC, ^13^C spectrum of cell suspension after approximately 18 s, and integrals of hyperpolarized αKIC and leucine as a function of time. **A.** αKIC is metabolized to leucine by BCAT. *T*_1_ times of hyperpolarized αKIC in H_2_O and D_2_O at 7 T are shown below αKIC. Interconverted functional groups are marked in orange. Glu: glutamate; αKG: α-ketoglutarate. **B.** ^13^C NMR spectra were recorded as a pseudo-2D experiment every 3 s. Intensity relative to the highest αKIC signal. **C.** 7^th 13^C NMR spectrum with detectable signal. Intensity relative to the highest αKIC signal. **D.** Analytic “fit” described by Khegai et al. is shown as an orange line.^57^ Leucine integrals scaled by a factor of 97 for visibility.

As a proof of principle study of this new PHIP-SAH probe, we conducted *in vitro* cell experiments to monitor the biorelevant, enzymatic conversion of αKIC to the essential amino acid leucine by BCAT. Our goal was to demonstrate the ability to observe real-time conversion and obtain kinetic rates as a potential metric for BCAT activity. For our study, we used a variety of human Burkitt lymphoma (RAMOS and DG-75) and human T cell leukemia cells (JURKAT), all of which showed sufficient conversion, visible at an emerging peak at 176.3 ppm (see Figures 3 **B** and **C** and S4 in the Supporting Information). Notably, the buildup of leucine to a maximum after approximately 15 s post-initial signal detection can be observed, which is crucial for calculating reliable metabolic flux. To estimate the rate of the αKIC-to-leucine conversion, we analyzed the obtained kinetic data according to the procedure described by Khegai et al.^57^ and calculated metabolic flux values (see Figure 3 **D** and Supporting Information). The production of [1-^13^C]­leucine-*d*_3_ and [1-^13^C]­leucine-*d*_2_ from hyperpolarized αKIC by the used cancer cells was independently confirmed by mass spectrometric analysis of the cells and the cell suspension supernatant (see Supporting Information).

For a clinical translation of parahydrogen-based approaches, toxicity studies need to be conducted to determine exact residual compounds (including catalyst, solvents, etc.) and define safe levels for regulatory approval. Generally, ICH guidelines stipulate exposure to less than 10 μg of rhodium and 50 mg of organic solvents (class 3; such as acetone and ethanol) per day for injection.^58,59^ Steps have been published in the literature toward this direction, showing low levels of rhodium and solvents.^18^ Specifically for this work, residual ethanol levels from side arm cleavage can be estimated to be below 1 mg, even at the higher concentrations reported (only ∼0.6 mg EtOH-*d*_3_ should be produced from 0.2 mL of 68 mM VαKIC samples before evaporation). Successful scale-up attempts of the PHIP-SAH technique for preclinical applications (70–160 mM hyperpolarized metabolite, 2 mL) have also been reported and extensively studied, delivering similar results to dDNP.^18,60^ That the ICH limits can also be met with parahydrogen-based approaches in volumes of 30–50 mL^61^ would be a great step toward regulatory approval. Finally, a sterile and pyrogen-free production needs to be ensured in this process.

## Conclusion

In this work, we report a new, reliable synthesis for obtaining vinyl esters of α-ketoacids via a metal-catalyzed gas/liquid reaction. This reaction was employed in a two-step synthesis to obtain vinyl-*d*_3_ [1-^13^C]­α-ketoisocaproic acid-3,3-*d*_2_ (VαKIC), a new metabolic precursor for hyperpolarized MR, with a good overall yield of 41–45%. Using the PHIP-SAH approach, we demonstrate quick and reliable production of high ^13^C polarization levels, competitive with those reported for dDNP:^37^ 39% (4.1 mM) and 27% (19 mM) for biocompatible αKIC, suitable for *in vitro* cell studies and potential initial preclinical *in vivo* studies, respectively. Nevertheless, further upscaling of the sample concentration and volume is necessary for translation into clinical applications, which typically use concentrations of ∼250 mM of the hyperpolarized metabolic tracer. With initial *in vitro* cell experiments in three human cancer cell lines, we further prove biocompatibility, having removed organic solvents (acetone-*d*_6_ and ethanol, from side arm cleavage) and the catalyst within seconds. More importantly, these studies demonstrate sufficient metabolic conversion to leucine to obtain reliable kinetic rates as a potential metric for BCAT activity, making this a relevant development toward broadening metabolic disease monitoring.

## Supplementary Material



## Notes

Further instrumentation and analysis information, discussion of reaction mechanism, application of hyperpolarized EtαKIC for *in vitro* studies, *T*_1_ determination, additional cell data, NMR spectra, and additional references (Refs 62–77 ) (PDF)
