# Supplementary material for: Purified [1-13C]α-Ketoisocaproate Spin-Polarized Up to 39% with Parahydrogen Enables Real-Time Metabolism Studies in Cancer Cells
Source: ACS Meas Sci Au. 2026 Aug 17;6(5):1561–70. doi: 10.1021/acsmeasuresciau.6c00117 (PMC13693266; doi:10.1021/acsmeasuresciau.6c00117)
Supplement: Supplementary file 1 [file tg6c00117_si_001.pdf]

Supporting Information

Purified [1-^{13}C]α-Ketoisocaproate spin-polarized up to 39% with parahydrogen enables real-time metabolism studies in cancer cells

Leander K. May, Lena Börschmann, Denis Moll, Lisa M. Fries, Gonzalo G. Rodriguez, Charlotte von Petersdorff-Campen, Maria D. Santi, Sergey Korchak, Andrei Leonov, Patrick Giavalisco and Stefan Glöggler*

[a] L. K. May, L. Börschmann, Dr. D. Moll, Dr. L. M. Fries, Dr. G. G. Rodriguez, C. von Petersdorff-Campen, Dr. M. D. Santi, Dr. S. Korchak, Prof. Dr. S. Glöggler
NMR Signal Enhancement
Max Planck Institute for Multidisciplinary Sciences
Am Fassberg 11, Göttingen 37077, Germany

[b] Center for Biostructural Imaging of Neurodegeneration of the University Medical Center
Von-Siebold-Str. 3a, Göttingen 37075, Germany

[c] Dr. A. Leonov
NMR-based Structural Biology
Max Planck Institute for Multidisciplinary Sciences
Am Fassberg 11, Göttingen 37077, Germany

[d] Dr. P. Giavalisco
Metabolomics Core Facility
Max Planck Institute for Biology of Ageing
Joseph Stelzmann Str. 9b, Cologne 50931, Germany

[e] Prof. Dr. S. Glöggler
Advanced Imaging Research Center
University of Texas Southwestern Medical Center
Dallas, Texas, 75390, USA

[f] Department of Biomedical Engineering
University of Texas Southwestern Medical Center
Dallas, Texas, 75390, USA
E-mail: stefan.gloeggler@UTSouthwestern.edu

Contents

Contents 2
NMR Spectroscopy 3
Mass Spectrometry 3
Statistical Analysis 3
Determination Degree of Deuteration 3
Discussion of Reaction Mechanism of Vinylation Reaction 4
Quantification of ^{13}C Polarization 6
Application of Hyperpolarized EtαKIC for *In Vitro* Studies 7
T_1 Determination 8
Additional Information of PHIP Experiments in Cells 10
Kinetic Model and Cell Data Analysis 12
Khegai Model 12
Results Data Analysis and Visualization 14
RAMOS 16
DG-75 17
JURKAT 18
Spectra 19
References 25

NMR Spectroscopy

^{1}H, ^{2}H and ^{13}C NMR spectra were recorded on a *300 MHz Bruker UltraShield™ Avance™ III HD* or a *300 MHz Bruker Ascend™ WB Avance™ NEO* spectrometer at 25 °C. Chemical shifts are reported in ppm relative to tetramethylsilane. ^{1}H NMR spectra were referenced to the residual proteosolvent peak respectively according to Table S1. Multiplicities are shown as s (singlet), d (doublet), t (triplet), q (quartet), quin. (quintet), sext. (sextet), sept. (septet), non. (nonet), ap. (apparent), br. (broad) or m (multiplet). Coupling constants, *J*, are reported in Hertz (Hz), rounded to the nearest 0.1 Hz and as observed. Integration of peaks is provided with the assignments indicated where appropriate. If not stated otherwise, ^{13}C spectra were recorded with ^{1}H and ^{2}H decoupling during acquisition.

Table S1 ^{1}H NMR chemical shift of residual proteosolvent peak and ^{13}C NMR chemical shift of solvent peak.

Solvent	δ^{1}_{H} / ppm	δ^{13}_{C} / ppm
$CDCl_3$	7.26	77.16
Acetone-d_6	2.05	29.84, 206.26
D_2O	4.79	-

Mass Spectrometry

All recorded spectra were measured by the *Central Analytics / Mass Spectrometry* department of the *Faculty of Chemistry* of the *Georg August University of Göttingen.* Spectra were recorded using a *Thermo Scientific™ Orbitrap Exploris™ 120* instrument. Dimensionless mass-to-charge ratios (*m/z*) are given.

Statistical Analysis

Statistical analysis was done using *GraphPad Prism 10.4.1 (627)*[1]. For the analyses unpaired one-tailed *t*-tests were performed. Gaussian distribution and identical standard deviations were assumed in all cases. ns: $P > 0.05$; *: $P \leq 0.05$; **: $P \leq 0.01$; ***: $P \leq 0.001$; ****: $P \leq 0.0001$.

Determination Degree of Deuteration

The degree of deuteration was determined using either an internal or external standard (see Equation 1). When using an internal standard, the integral of the deuterated position is compared to an undeuterated position in the molecule to give a conservative estimate of the degree of deuteration. For external standards, NMR experiments were performed under identical conditions (number of scans, receiver gain, sample volume, temperature).

$$D = 100 - \left(100 * \frac{I_{\text{analyte}}}{I_{\text{reference}}}\right) \quad (1)$$

Discussion of Reaction Mechanism of Vinylation Reaction

Our proposed mechanisms are based on the iridium-catalyzed intramolecular hydroamination reported by Hesp *et al.*[2], who discussed two possible mechanisms by means of computational chemistry. In their study, they concluded that the π-acid catalyzed cyclisation was favored over the amine activation as the initial activation of the N–H bond showed a rather high reaction barrier of 38.3 kcal mol^{-1}, compared to the activation barrier of 16.9 kcal mol^{-1} for the nucleophilic attack on the olefin, thus being in disagreement with their observation of a smooth reaction progression. As the operative mechanism, they suggested the initial coordination of the olefin to obtain the catalytically active, monomeric, electron-poor iridium(I) species ([Ir(COD)Cl(substrate)]), which subsequently undergoes nucleophilic attack of the amine on the olefin. After proton transfer from the amine to the iridium, the product is then obtained through a reductive elimination.

Given the similarity of the catalyst and the substrates, a similar high activation barrier for the activation of the O–D bond can be expected, as the addition of the formal deuteron via oxidation of the iridium(I) to iridium(III) should be limiting in both cases (electronic and structural rearrangement of the metal center should show a high energy barrier[3]). Since the vinylation is performed at relatively low temperature (48 °C), compared to the intramolecular hydroamination (110 °C), the activation energy necessary for O–D bond cleavage and addition step is even less likely to be given, thus rendering the π-acid catalyzed mechanism more likely to be operative.

This is in agreement with our observations of an increased reaction rate upon addition of catalytic amounts of base (10 mol% Na_2CO_3), thus favoring the formation of the carboxylate for the nucleophilic attack on the acetylene-d_2. The observation of product formation even in absence of base leads to the suggestion that either autoprotolysis of the carboxylic acid is sufficient for reaction progression or that the second mechanism is competitive in absence of additional base. Additionally, Werner *et al.*[4] reported an iridium(III)-σ-alkenyl species for the reaction of an activated carboxylic acid (F_3CCO_2H) with an iridium(I) species, which is reminiscent of the proposed intermediate of the O–D bond activation mechanism.

Isotope labelling experiments with undeuterated αKIC and acetylene-d_2 show a low incorporation of deuterium on all vinyl positions (D_{vinyl} = 69 – 82%), suggesting an isotope exchange of acetylene-d_2 before or during the vinylation reaction. This can be rationalized by a deprotonation/protonation mechanism of acetylene-d_2 taking place at the iridium(I) center, resulting in a semi-statistical distribution of isotopolouges of acetylene: acetylene-d_2, acetylene-d_1 and acetylene-d_0 (see Scheme S1). Such a depotonation/protonation reaction with rhodium and phenylacetylene, as well as the H/D exchange of an iridium(III)-σ-alkinyl species were previously described by Werner *et al.*[5] and Höhn *et al.*[6], respectively. Höhn *et al.*[6] mixed [Ir**H**(C_2Me)Cl(P^iPr_3)$_2$] with [Ir**D**(C_2Ph)Cl(P^iPr_3)$_2$] and could observe the exchange product [Ir**H**(C_2Ph)Cl(P^iPr_3)$_2$] within minutes, suggesting a rapid isotope exchange. Additionally, similar complexes to the iridium(III)-σ-alkinyl starting from iridium(I) and acetylene derivatives have been reported previously by Werner *et al.*[4] and Höhn *et al.*[6] experimentally (no direct reaction of acetylene with the iridium catalyst could be observed in [6], but the structural iridium(III)-σ-alkinyl was shown via another synthetic route) and by Wang *et al.*[7] computationally.

Analogous to this deprotonation/protonation reaction, a migratory insertion/β-deuteride elimination mechanism could be proposed for the isotope scrambling in case of an operative O–D bond activation mechanism. Migratory insertion reactions of acetylene derivatives are known for many late transition metals (including iridium)[8-13], but to our knowledge, no beta-hydride elimination from a vinylidene to an acetylene was reported previously. Nevertheless, to comply with microscopic reversibility, this step has to be considered.

Additional deuteration of the β-position of about 77% could also be observed and leads to the suggestion of further reactivity of the metal catalyst with acetylene-d_2 in isotope scrambling reactions. The participation

of the solvent or other proton sources in the isotope scrambling can be neglected, as otherwise a significant degree of protonation should be observed in case of the reaction of [1-^{13}C]αKIC-d_3 and acetylene-d_2, which is not the case.

Using the sodium salt of αKIC resulted in no product formation, proving the importance of the free carboxylic acid moiety in the reaction (i.e. for deuterodemetallation or migratory insertion into the Ir–D bond) and the inability of other compounds (i.e. DCM) to participate in this reaction.

Since the focus of this communication is presenting the developed reaction and using the synthesized compounds for hyperpolarization via PHIP-SAH, additional mechanistic experiments or computational analyses to elucidate the exact reaction mechanism were not performed. Furthermore, trapping experiments of catalytic species are difficult to perform as the reaction is typically conducted under elevated pressure.

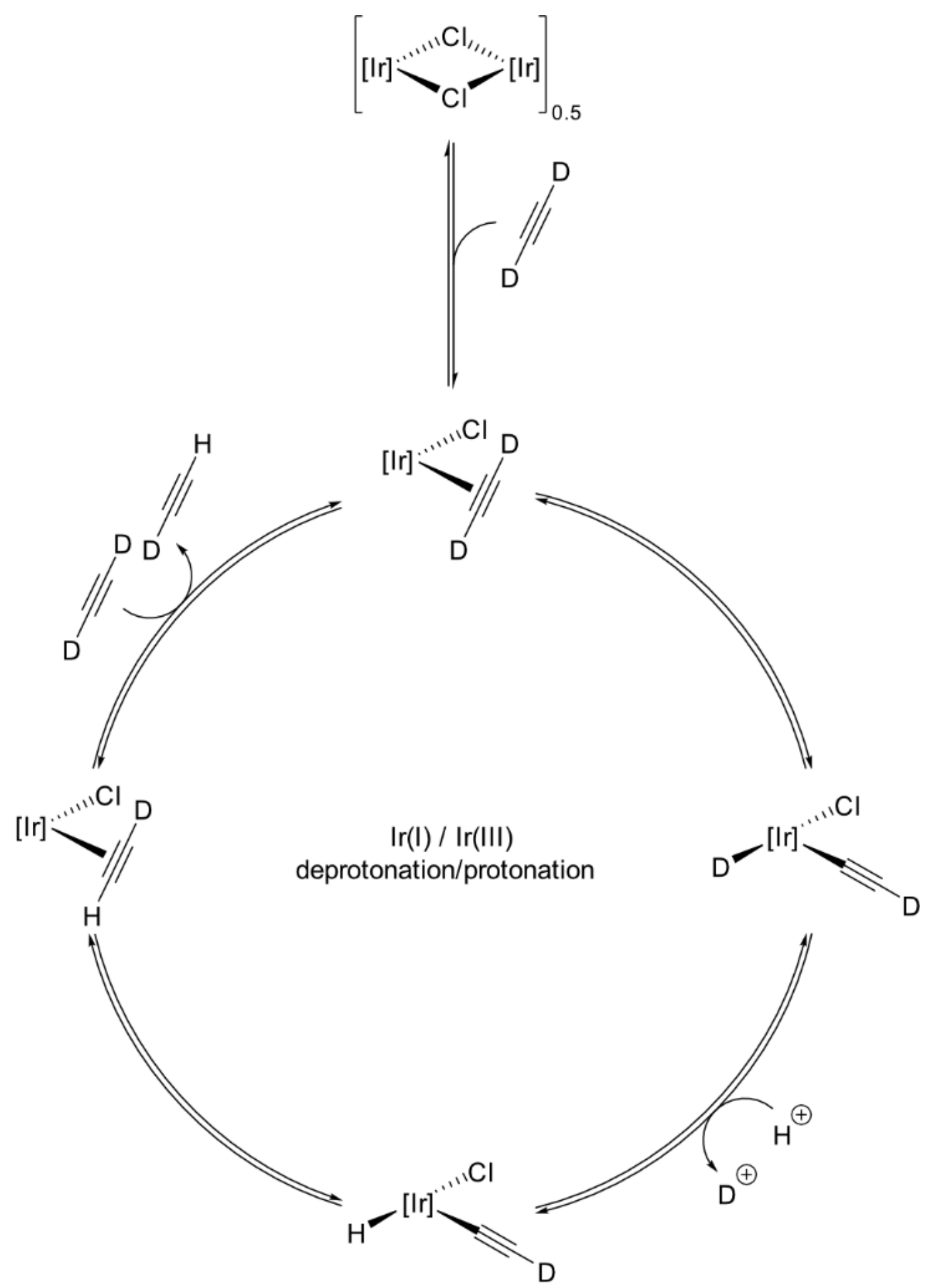

Scheme S1 Proposed mechanism of the isotope scrambling of acetylene-d_2. Iridium(I) catalyzed isotope exchange of acetylene-d_2 resulting in a semi-statistical distribution of acetylene-d_2, acetylene-d_1 and acetylene-d_0. The exact coordination sphere of the iridium center is not discussed and is abbreviated as [Ir].

Quantification of ^{13}C Polarization

To quantify the ^{13}C polarization after the PHIP experiment, a thermal ^{1}H NMR spectrum was measured immediately afterwards to determine the EtαKIC or αKIC concentration (w/o workup: within 1 min; w/ workup: within 5 – 10 min after hyperpolarization). If no subsequent workup was performed, a pressure of 8 bar was maintained using N_2. The concentration of the sample was determined from the thermal spectrum using the Me-groups (w/o workup: 0.985 – 1.150 ppm (3.4 mM) and 0.900 – 1.200 ppm (13 mM); w/ workup: 1.050 – 1.250 ppm (3.4 mM), 0.900 – 1.200 ppm (13 mM) and 0.900 – 1.300 ppm (68 mM)). The ^{13}C polarization (P_{hyper}) was then calculated using a thermal reference spectrum (see Table 1; 0.5 M sodium [1-^{13}C]pyruvate; 0.2 mL D_2O, 298K; n_{thermal} = 4; w/o workup: no ^{1}H and ^{2}H decoupling during acquisition; w/ workup: ^{1}H and ^{2}H decoupling during acquisition) according to Equations 2 and 3. Complete hydrogenation of the starting material was assumed in all cases.

Table S1 Average integral of thermal ^{13}C reference spectra (n = 3) of 0.5 M sodium [1-^{13}C]pyruvate (I_{thermal}) without and with ^{1}H and ^{2}H decoupling during acquisition. Sample volume: 0.2 mL D_2O; n_{thermal} = 4.

NMR experiment	I_{thermal} / a.u.	NMR experiment	I_{thermal} / a.u.
^{13}C	2 755 968.16	$^{13}C\{^{1}H, ^{2}H\}$	3 483 477.03

$$P_{\text{hyper}} = \frac{I_{\text{hyper}}}{I_{\text{thermal}}} \cdot \frac{n_{\text{thermal}}}{n_{\text{hyper}}} \cdot \frac{c_{\text{thermal}}}{c_{\text{hyper}}} \cdot \frac{\sin(\theta_{\text{thermal}})}{\sin(\theta_{\text{hyper}})} \cdot P_{\text{thermal}} = \varepsilon \cdot P_{\text{thermal}} \tag{2}$$

$$P_{\text{thermal}} = \tanh\left(\frac{\hbar\gamma B_0}{2k_{\text{B}}T}\right) \approx 5.84 \cdot 10^{-6} \tag{3}$$

I_x represents the obtained integrals, n_x represents the number of scans (n_{hyper} = 1), c_x represents the concentration and Θ_x represents the flip angle ($\Theta_{\text{hyper}} = \Theta_{\text{thermal}}$ = 90 °) of the hyperpolarized and thermal spectra. The receiver gain (rg_X) was set the same for all experiments ($rg_{\text{hyper}} = rg_{\text{thermal}}$ = 10). For the thermal polarization P_{thermal}, the reduced Planck constant $\hbar$ (1.0545718 · 10^{-34} J s rad^{-1}), the gyromagnetic ratio γ (67.2828 · 10^{6} rad s^{-1} T^{-1}), the applied magnetic field B_0 (7.04950876 T), the Boltzmann constant k_B (1.38064853 · 10^{-23} J K^{-1}) and the temperature T were used. Additionally, the enhancement factor ε can be determined. It is defined as the product of all fractions in Equation 2.

Tables S2 – S6 show the data from the performed PHIP experiments.

Table S2 Integral of the ^{13}C NMR spectra (I_{hyper}), concentration of the sample (c_{hyper}) and calculated ^{13}C polarization of hyperpolarized EtαKIC (P_{hyper}) after the PHIP experiment and enhancement factor (ε) of 3.4 mM samples without workup at 320 K.

n	I_{hyper} / a.u.	c_{hyper} / mM	P_{hyper} / %	ε
1	622 266 818.91	3.38	75.63	133 603
2	619 362 143.88	3.40	74.83	132 197
3	614 913 997.86	3.31	76.32	134 816
4	612 944 686.72	3.30	76.30	134 792
5	618 490 135.31	3.32	76.53	135 192
6	632 242 899.97	3.63	71.55	126 396

Table S3 Integral of the $^{13}C\{^{1}H, ^{2}H\}$ NMR spectra (I_{hyper}), concentration of the sample (c_{hyper}) and calculated ^{13}C polarization of hyperpolarized αKIC (P_{hyper}) after the PHIP experiment and enhancement factor (ε) of 3.4 mM samples after workup at 320 K.

n	I_{hyper} / a.u.	c / mM	P_{hyper} / %	pH	ε
1	134 299 733.33	1.17	37.31	7.5	65 903
2	108 161 571.56	0.83	42.35	7.0	74 819
3	137 361 016.19	1.35	33.07	8.5	58 418
4	122 315 641.34	1.53	25.98	8.5	45 899

Table S4 Integral of the ^{13}C NMR spectra (I_{hyper}), concentration of the sample (c_{hyper}) and calculated ^{13}C polarization of hyperpolarized EtαKIC (P_{hyper}) after the PHIP experiment and enhancement factor (ε) of 13 mM samples without workup at 310 K.

n	I_{hyper} / a.u.	*c* / mM	P_{hyper} / %	ε
1	2 004 105 193.44	12.37	68.70	117 573
2	2 048 141 675.31	11.05	78.60	134 510
3	2 106 666 144.78	12.32	72.51	124 091
4	2 090 271 060.81	12.67	69.96	119 724

Table S5 Integral of the $^{13}C\{^1H, ^2H\}$ NMR spectra (I_{hyper}), concentration of the sample (c_{hyper}) and calculated ^{13}C polarization of hyperpolarized αKIC (P_{hyper}) after the PHIP experiment and enhancement factor (ε) of 13 mM samples after workup at 310 K.

n	I_{hyper} / a.u.	*c* / mM	P_{hyper} / %	pH	ε
1	470 544 959.75	4.06	38.88	7.0	66 541
2	536 994 014.74	4.30	41.90	7.5	71 700
3	433 000 834.28	3.99	36.41	7.5	62 306

Table S6 Integral of the $^{13}C\{^1H, ^2H\}$ NMR spectra (I_{hyper}), concentration of the sample (c_{hyper}) and calculated ^{13}C polarization of hyperpolarized αKIC (P_{hyper}) after the PHIP experiment and enhancement factor (ε) of 68 mM samples after workup at 320 K.

n	I_{hyper} / a.u.	*c* / mM	P_{hyper} / %	pH	ε
1	1 568 290 518.36	21.52	23.69	7.0	41 841
2	1 553 862 787.05	16.21	31.15	7.0	55 036
3	1 641 257 737.31	19.64	27.16	7.0	47 979

Application of Hyperpolarized EtαKIC for *In Vitro* Studies

The utility of the hyperpolarized ethyl derivative, EtαKIC, for potential improved cell permeability in later studies was also investigated. For this purpose, the workup procedure was adjusted to substitute the addition of base with addition of buffer (2 x 100 μL buffer instead of 100 μL base + 100 μL buffer). This resulted in two small signals of αKIC and EtαKIC (160.2 ppm) in a ratio of about 1 to 0.6 – 2.5, respectively, due to the onset of sidearm cleavage. The use of EtαKIC for *in vitro* or *in vivo* experiments is therefore expected to be restricted.

T_1 Determination

The T_1 times of the hyperpolarized precursor (EtαKIC) and the free αKIC after workup were determined in acetone-d_6 and deuterated and non-deuterated buffer solutions, respectively, for at least three low concentration samples. For this, ^{13}C NMR spectra were acquired with a repetition time of t_r = 10 s using Θ = 18° pulses in a pseudo-2D sequence. The respective peaks were integrated and normalized to the maximum signal of each dataset. The data were fitted with an exponential decay function providing an apparent rate constant R_1', which was corrected for the decrease in magnetization caused by repeated pulses (Equations 4 and 5).

$$R_1 = R_1' + \frac{\ln\left(\cos(\theta)\right)}{t_\mathrm{r}} \quad (4)$$

$$T_1 = \frac{1}{R_1} \quad (5)$$

The respective plots with the mean-squared error (*MSE*) of the fit can be found in Figures S1 – S3.

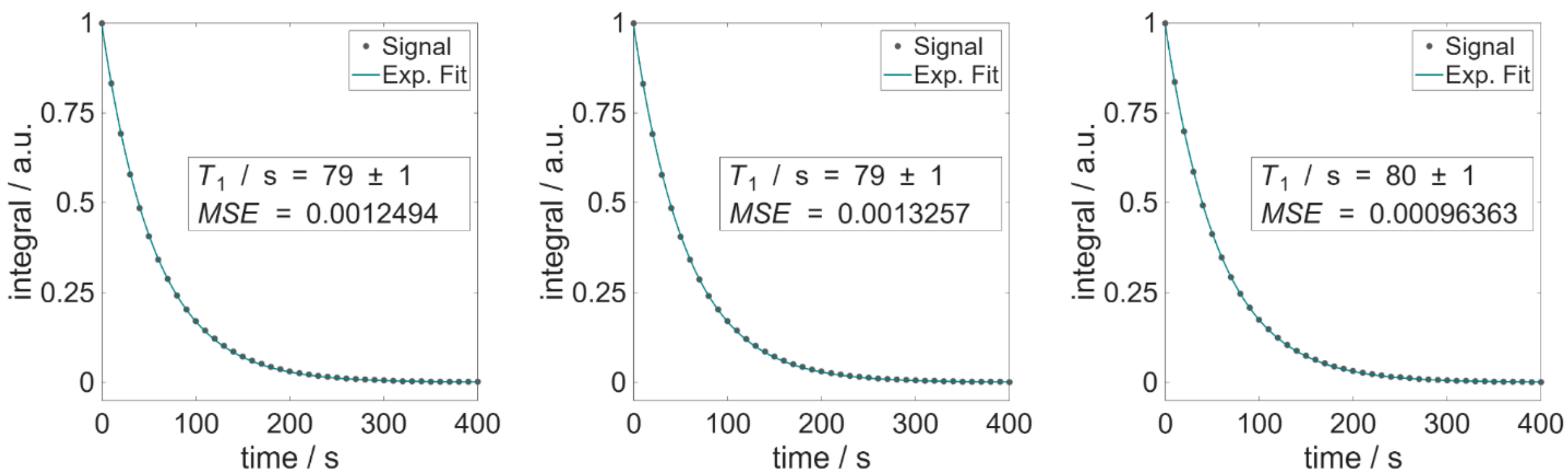

Figure S1 T_1 measurements of EtαKIC at 7 T in acetone-d_6.

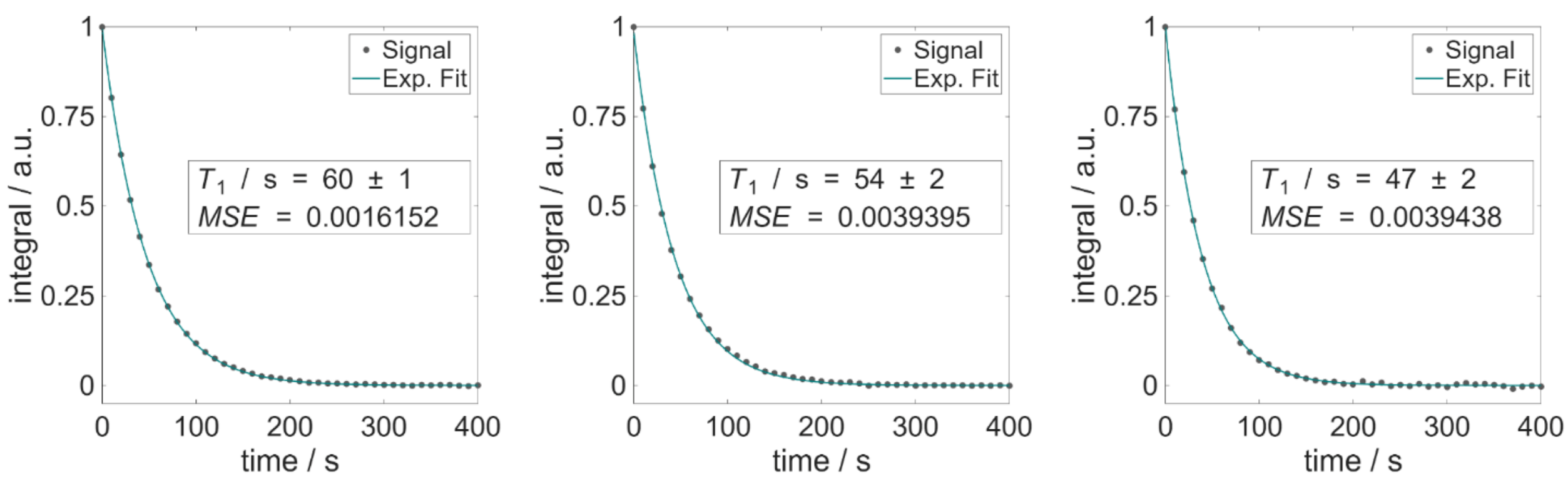

Figure S2 T_1 measurements of αKIC at 7 T in non-deuterated buffer.

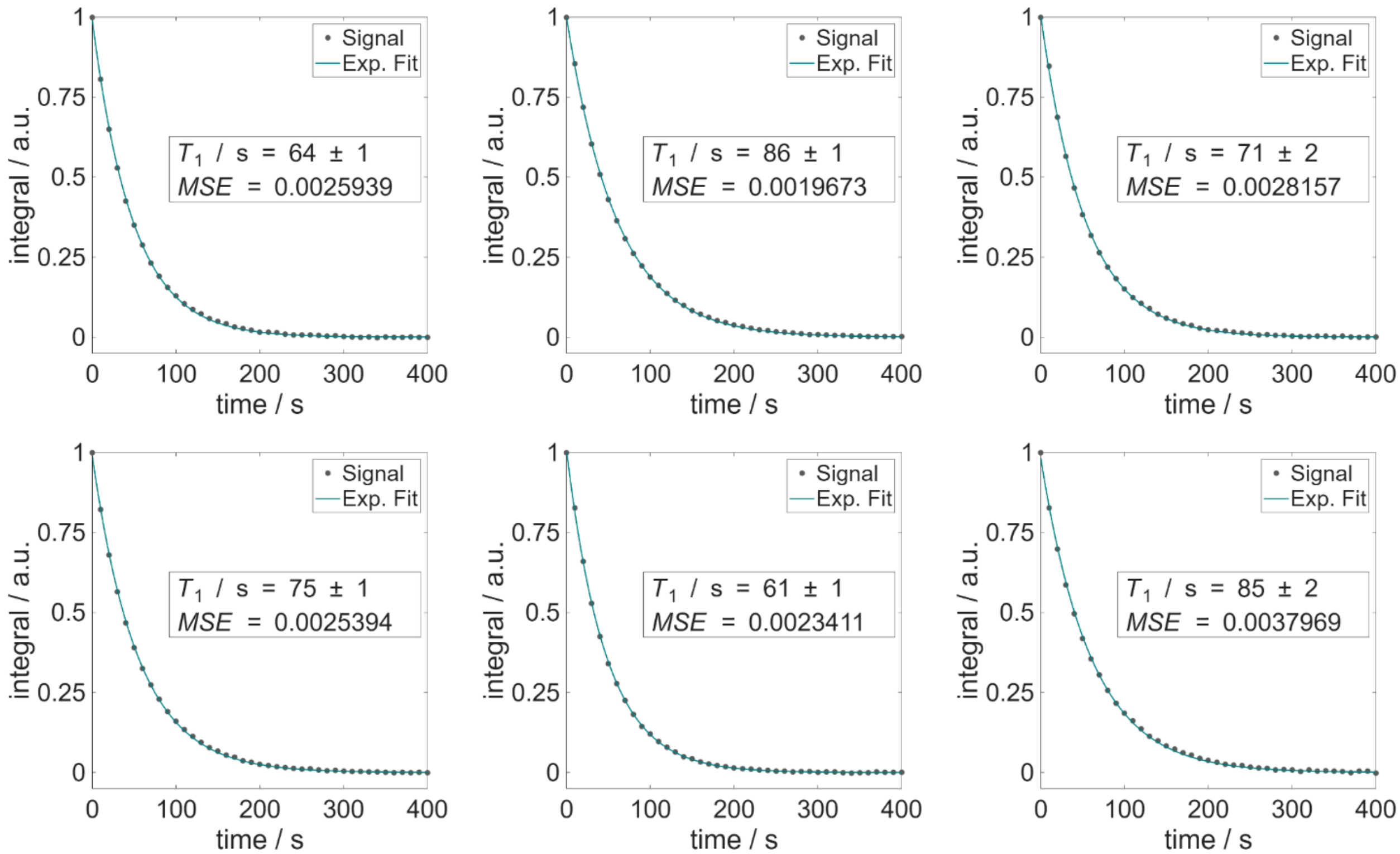

Figure S3 T_1 measurements of αKIC at 7 T in deuterated buffer.

Additional Information of PHIP Experiments in Cells

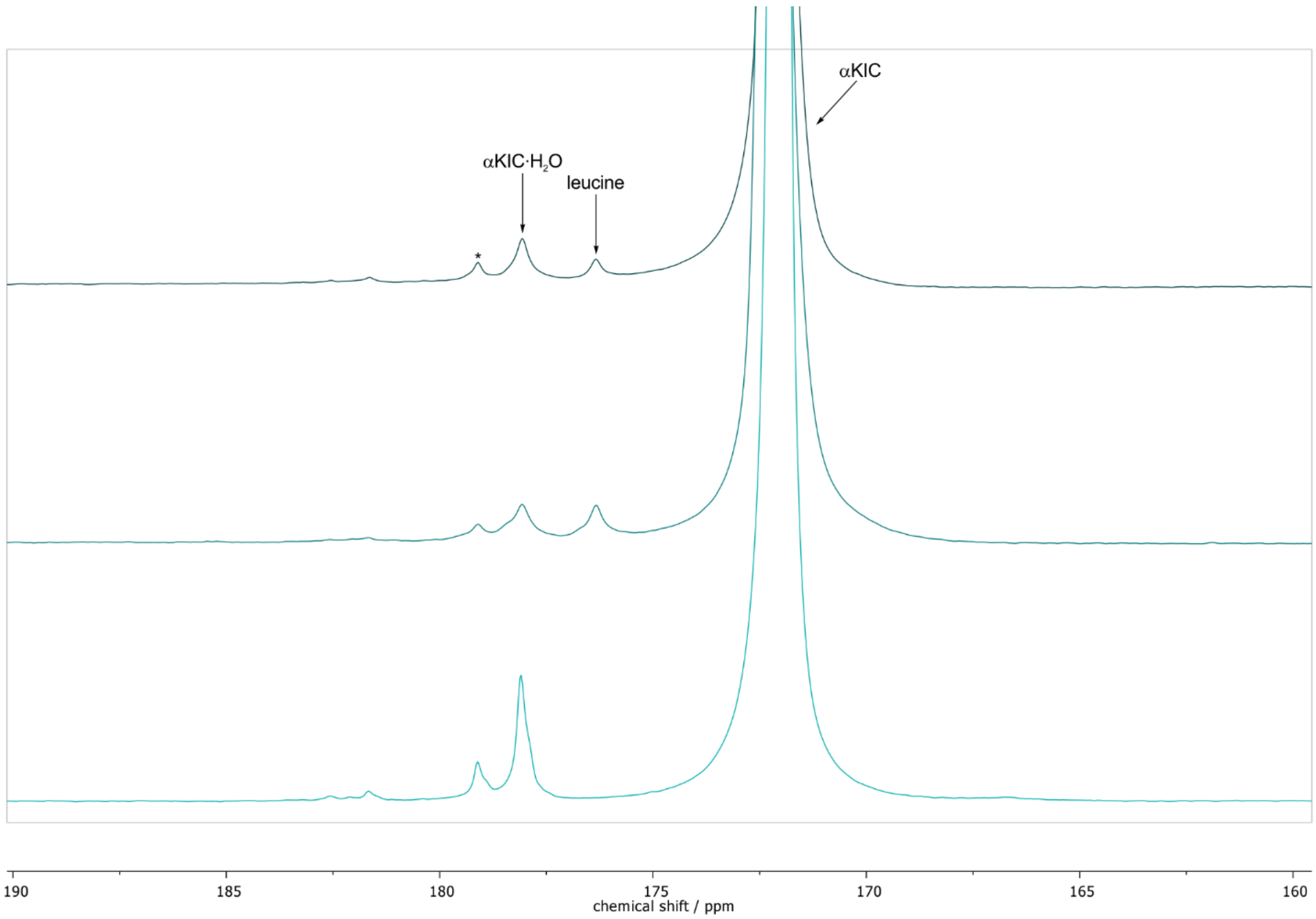

Figure S4 Representative summed ^{13}C spectra of cell experiments 1 – 3. Normal procedure (cells + medium with 10% FBS + VαKIC, top), procedure without FBS (cells + medium without FBS + VαKIC, mid) and procedure without cells (medium with 10% FBS + VαKIC, bottom). αKIC (172.1 ppm), leucine (176.3 ppm) and αKIC·hydrate (~178.1 ppm) peaks marked with arrow. Impurity marked with asterisk.

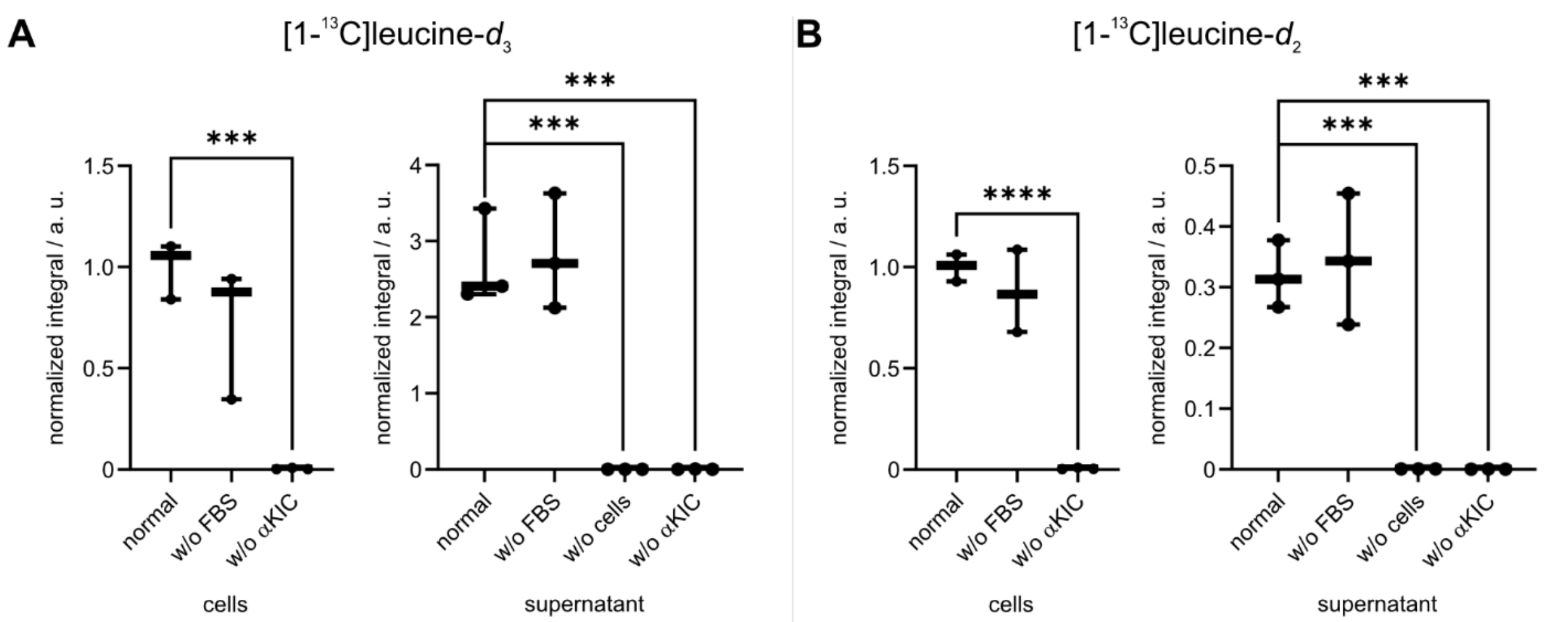

Figure S5 Comparison of [1-^{13}C]leucine-d_3 and [1-^{13}C]leucine-d_2 amounts of experiments 1 – 4 intracellularly and in the supernatant of the cell suspension. **A** Relative amount of [1-^{13}C]leucine-d_3 intracellularly (left) and in the supernatant (right). **B** Relative amount of [1-^{13}C]leucine-d_2 intracellularly (left) and in the supernatant (right). Integrals normalized to the average amount of [1-^{13}C]leucine-d_3 and [1-^{13}C]leucine-d_2 found in the cells using the normal procedure. ns: $P > 0.05$; *: $P \leq 0.05$; **: $P \leq 0.01$; ***: $P \leq 0.001$; ****: $P \leq 0.0001$.

Mass spectrometric analysis revealed higher levels of [1-^{13}C]leucine-d_3 and [1-^{13}C]leucine-d_2 intracellularly and in the supernatants for experiments 1 (normal procedure) and 2 (without FBS) compared to control experiments 3 (without cells) and 4 (without VαKIC), which did not involve addition of cells or hyperpolarized αKIC (see Figure S5). These results align with our observations during the PHIP experiments, in which JURKAT cancer cells actively converted hyperpolarized αKIC to leucine. No significant influence of FBS could be observed.

Kinetic Model and Cell Data Analysis

^{13}C NMR spectra were processed using *Bruker TopSpin 4.5.0*[14] Phase corrections in the 0th order, line broadening to 10 Hz and baseline corrections were applied. Kinetic analysis was performed and plotted using *Matlab R2025a*[15]. For the analysis of the kinetics of the αKIC-to-leucine conversion the two-way system, proposed by Khegai *et al.*[16], in which all sources for leucine signal loss are summarized in an effective relaxation rate R_{eff}, was considered (see Scheme S2).

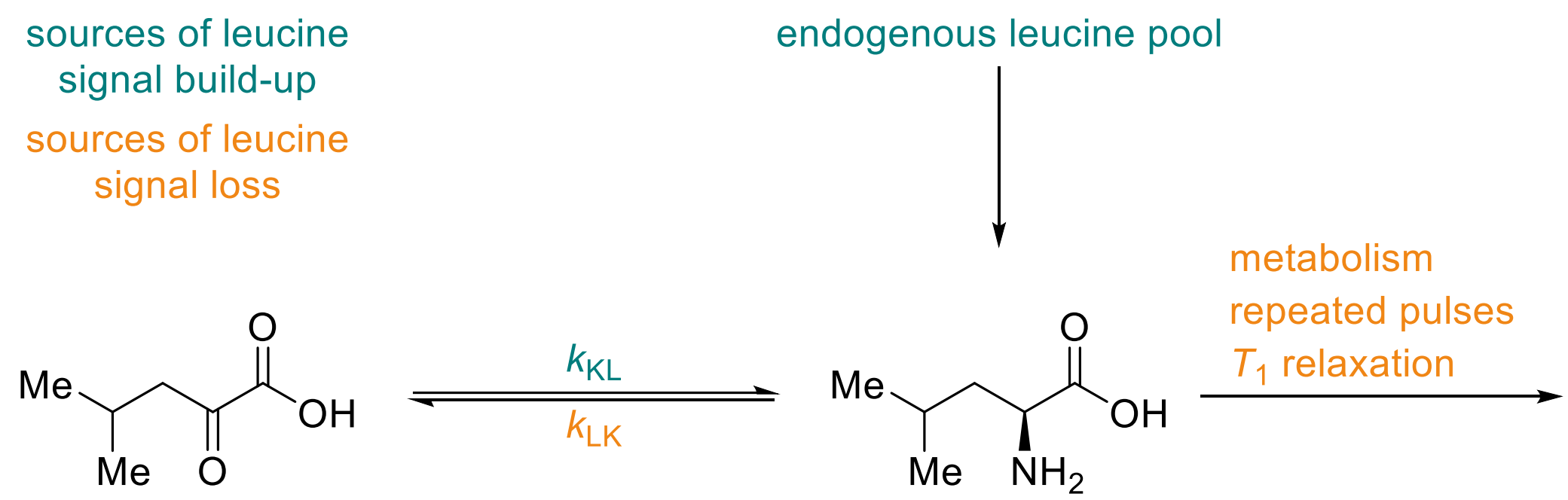

Scheme S2 Schematic depiction of the two-way model for the conversion of αKIC to leucine. Sources of leucine signal build-up shown in cyan; sources of leucine signal loss shown in orange.

Khegai Model

The individual αKIC (172.1 ppm) and leucine (176.3 ppm) integrals were obtained by fitting the acquired spectra by two Lorentzian functions, as proposed by Khegai *et al.*[16]. Firstly, the dominant peak (αKIC) was fitted by a single Lorentzian function. Secondly, the residual spectrum, i.e. the original spectrum minus the fitted αKIC Lorentzian, was fitted by the second Lorentzian and associated with leucine. In case no peak could be observed, the integral was set to 0 and not included for the Khegai fitting of leucine. The two signals were then integrated, keeping the integration limits on ± 5 times the full width at half prominence of each Lorentzian function. A baseline correction was performed on all spectra based on the baseline of the last spectrum before initial signal detection in the dataset (no αKIC or leucine signal). The first ^{13}C spectrum (in some cases the first two) was disregarded due to inconsistencies with the visualization of the calculated leucine data (calculated leucine data is discussed below). Integrals were normalized to the signal of highest intensity in the dataset, which corresponded to the first αKIC integral in most cases. The leucine integrals were smoothed using the "rloess" method of the "smoothdata" function from *Matlab*, as some datasets showed noisy leucine integrals given the low amplitude of its peaks. This data processing has resulted in more accurate and robust conversion rate calculations, as described below.

To estimate the αKIC-to-leucine conversion of the injected hyperpolarized αKIC, the rate constant k_{KL} was derived from the formulation of the two-way exchange model by Khegai *et al.*[16], with *KIC*(*t*) and *Leu*(*t*) the integrals of αKIC and leucine at time *t*, respectively.

$$\frac{dLeu(t)}{dt} = k_{\mathrm{KL}} \cdot KIC(t) - R_{\mathrm{eff}} \cdot Leu(t) \tag{6}$$

The finite, time-discretized formulation leads to an overdetermined, linear equation system, which can be transformed into an equation system and solved for k_{KL} and R_{eff} using the pseudoinverse.

$$\frac{\Delta Leu_i}{\Delta t} = k_{\mathrm{KL}} \cdot KIC_i - R_{\mathrm{eff}} \cdot Leu_i \tag{7}$$

$$\begin{pmatrix} \frac{Leu_2 - Leu_1}{t_\mathrm{r}} \\ \vdots \\ \frac{Leu_i - Leu_{i-1}}{t_\mathrm{r}} \end{pmatrix} = \begin{pmatrix} KIC_1 & -Leu_1 \\ \vdots & \vdots \\ KIC_{i-1} & -Leu_{i-1} \end{pmatrix} \cdot \begin{pmatrix} k_\mathrm{KL} \\ R_\mathrm{eff} \end{pmatrix} \quad (8)$$

$$\begin{pmatrix} k_\mathrm{KL} \\ R_\mathrm{eff} \end{pmatrix} = \begin{pmatrix} \frac{Leu_2 - Leu_1}{t_\mathrm{r}} \\ \vdots \\ \frac{Leu_i - Leu_{i-1}}{t_\mathrm{r}} \end{pmatrix} \cdot \begin{pmatrix} KIC_1 & -Leu_1 \\ \vdots & \vdots \\ KIC_{i-1} & -Leu_{i-1} \end{pmatrix}^+ \quad (9)$$

For ΔLeu_i the difference between two consecutive leucine integrals, Leu_i and Leu_{i-1}, at the ith and $(i-1)^{\mathrm{th}}$ timepoint and for Δt the repetition time t_r = 3 s between consecutive ^{13}C spectra were used.

In order to obtain the optimal fitting, several k_KL and R_eff values were calculated by starting from different time points (j = 1, 2, 3, …, 5). The accuracy of the calculated parameters was then measured using the mean-squared error (*MSE*), which involved back-calculating the leucine integrals using Equations 10 & 11 (and the calculated k_KL, R_eff, the complete set of experimental αKIC integrals, *KIC*, and the first experimentally determined leucine integral, Leu_i) over the complete dataset, and then comparing the results with the experimental leucine integrals, *Leu*. Calculations with the lowest *MSE* value were deemed best fitting and the respective k_KL and R_eff values used for further analysis. For visualization purposes, the best-fit calculated leucine integrals were plotted alongside the experimental αKIC and leucine integrals.

$$Leu_{j-1} = \frac{KIC_{j-1} \cdot R_\mathrm{eff} \cdot t_\mathrm{r} - Leu_j}{1 - k_\mathrm{KL} \cdot t_\mathrm{r}} \quad (10)$$

$$Leu_{j+1} = \left(\left(k_\mathrm{KL} \cdot KIC_j - R_\mathrm{eff} \cdot Leu_j \right) * t_\mathrm{r} \right) + Leu_j \quad (11)$$

Where Leu_{j-1} is used for the calculation of all data points before the start point j and Leu_{j+1} for all data points after the start point.

For the comparison of different experiments, the flux F_KL was calculated by normalizing the rate constant k_KL for the amount of αKIC (n_KIC) and the number of cells (*#Cells*) used in the experiment.

$$F_\mathrm{KL} = \frac{k_\mathrm{KL} \cdot n_\mathrm{KIC}}{\#Cells} \quad (12)$$

Results Data Analysis and Visualization

The calculated flux values F_{KL} are shown in Table S6. A small, but insignificant trend of the conversion of hyperpolarized αKIC to leucine in the different cell lines can be assumed from these flux values (RAMOS: 0.55, DG-75: 0.38 and JURKAT: 0.37 fmol $cell^{-1}$ min^{-1}). This trend of conversion in the RAMOS and JURKAT cell lines is consistent with the known transcription of BCAT in the literature.[17, 18] There was no transcription value found for the DG-75 cell line to be used for further comparison.

In the cell experiments, besides the expected peaks of αKIC (172.1 ppm), αKIC·hydrate (178.1 ppm) and leucine (176.3 ppm), small additional peaks at 179.1, 181.6 and 182.6 ppm were observed in the ^{13}C spectra of the kinetic data (approx. equal or lower intensity than the αKIC·hydrate). These peaks were also observed when using only buffer solution, without addition of the cell suspension, and were probably due to a small impurity in the VαKIC solution, since they were not observed when using a different batch of VαKIC in later experiments. Since these peaks were only observed in regions higher than 178.1 ppm (αKIC·hydrate), they did not affect the visibility of the leucine signal and the leucine signal was validated with mass spectrometry (see above).

Table S7 Amount of injected hyperpolarized αKIC (n_{KIC}), number of cells (*#Cells*), cell viability before and after PHIP experiments (*Viability*$_{Before}$ and *Viability*$_{After}$), calculated rate constants and relaxation rates (k_{KL} and R_{eff}) with mean-squared error (*MSE*) starting from different timepoints (*j* = 1, 2, 3, …, 5) and flux values (F_{KL}) for PHIP experiments performed with the RAMOS, DG-75 and JURKAT cell lines. Underlined values correspond to the best fit, as determined by the lowest *MSE* value. Asterisk denotes experiments in which the first two spectra were excluded due to poor phasing.

Cell line	*j*	n_{KIC} / µmol	*#Cells* / 10^6	*Viability*$_{Before}$ / %	*Viability*$_{After}$ / %	*t*	K_{KL} / s^{-1}	R_{eff} / s^{-1}	*MSE* / 10^6	F_{KL} / fmol $cell^{-1}$ min^{-1}
RAMOS	1	1.29	29.8	88.3	92.9	1	0.000238	0.052489	0.077999	0.856
						2	0.000250	0.054429	0.034580	
						3	0.000276	0.058338	0.033713	
						4	0.000302	0.062325	0.033104	
						5	0.000325	0.065617	0.023210	
	2	1.29	37.1	96.5	95.2	1	0.000150	0.044182	0.071759	0.501
						2	0.000165	0.047023	0.049172	
						3	0.000188	0.051207	0.039053	
						4	0.000212	0.055395	0.030522	
						5	0.000236	0.059388	0.027407	
	3	1.29	55.3	96.0	95.8	1	0.000166	0.043736	0.161265	0.322
						2	0.000179	0.045728	0.226017	
						3	0.000201	0.049055	0.121168	
						4	0.000227	0.052888	0.104987	
						5	0.000243	0.055195	0.139982	
	4	1.29	49.5	94.6	92.3	1	0.000287	0.049722	0.063243	0.532
						2	0.000299	0.051069	0.082143	
						3	0.000324	0.053841	0.063908	
						4	0.000340	0.055631	0.068439	
						5	0.000344	0.056065	0.058866	
DG-75	1*	1.29	50.5	99.6	99.1	1	0.000179	0.048232	0.066609	0.429
						2	0.000204	0.052142	0.036488	
						3	0.000232	0.056366	0.030532	
						4	0.000262	0.060670	0.027915	
						5	0.000284	0.063899	0.026030	
	2	1.29	44.8	99.1	98.1	1	0.000154	0.052362	0.066564	0.415
						2	0.000174	0.057256	0.041282	
						3	0.000199	0.063032	0.040756	
						4	0.000225	0.068634	0.028505	
						5	0.000244	0.072801	0.025863	
	3	1.29	60.4	98.8	97.5	1	0.000222	0.054533	0.104956	0.449
						2	0.000232	0.056167	0.075039	
						3	0.000267	0.061629	0.051803	
						4	0.000306	0.067622	0.047783	
						5	0.000349	0.073993	0.045927	
	4*	1.29	59.8	98.7	95.7	1	0.000107	0.039743	0.147480	0.220
						2	0.000123	0.043154	0.070724	
						3	0.000139	0.046613	0.071201	
						4	0.000157	0.050174	0.059298	
						5	0.000174	0.053481	0.057679	
JURKAT	1	1.29	36.9	99.3	98.9	1	0.000148	0.053656	0.102775	0.420
						2	0.000150	0.054138	0.044601	
						3	0.000173	0.059889	0.040171	
						4	0.000201	0.066446	0.035578	
						5	0.000233	0.073866	0.038852	
	2	1.29	56.8	97.9	95.4	1	0.000262	0.057210	0.180621	0.341
						2	0.000252	0.056175	0.094607	
						3	0.000249	0.055518	0.157874	
						4	0.000246	0.055110	0.146694	
						5	0.000236	0.053850	0.162526	
	3*	1.29	60.3	99.3	96.3	1	0.000271	0.062142	0.054359	0.347
						2	0.000266	0.061349	0.052581	
						3	0.000233	0.056149	0.068213	
						4	0.000164	0.045913	0.060164	
						5	0.000071	0.032814	0.160416	

RAMOS:

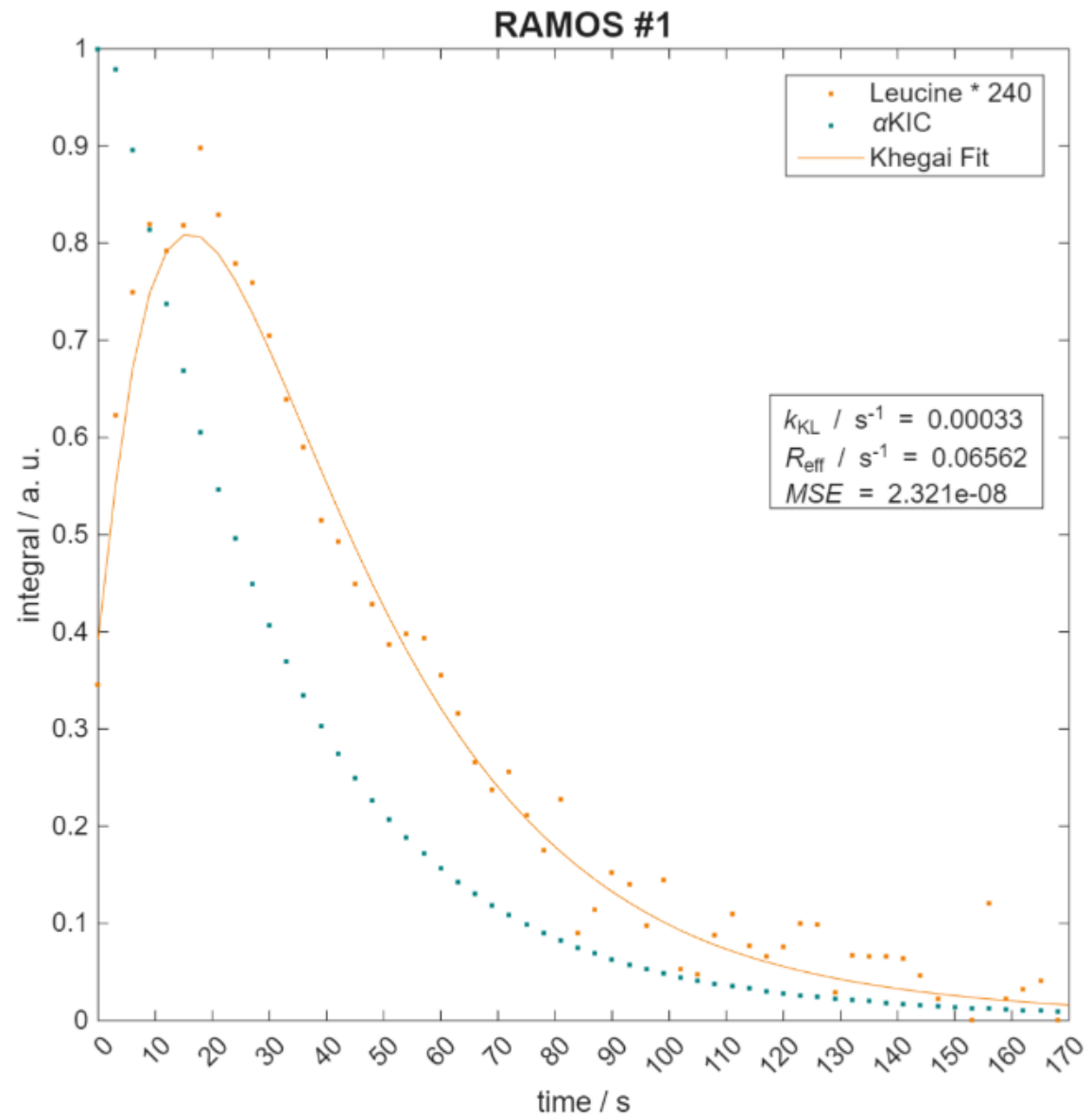

Figure S6 Normalized αKIC (cyan, dots), leucine (orange, dots), and recalculated leucine integrals (orange, line; here Khegai Fit) over time using the RAMOS cell line (*n* = 1). Leucine integrals scaled by a factor of 240 for visibility. Rate constant (k_{KL}), relaxation rate (R_{eff}) and mean-squared error (*MSE*) of the best fit are shown on the right.

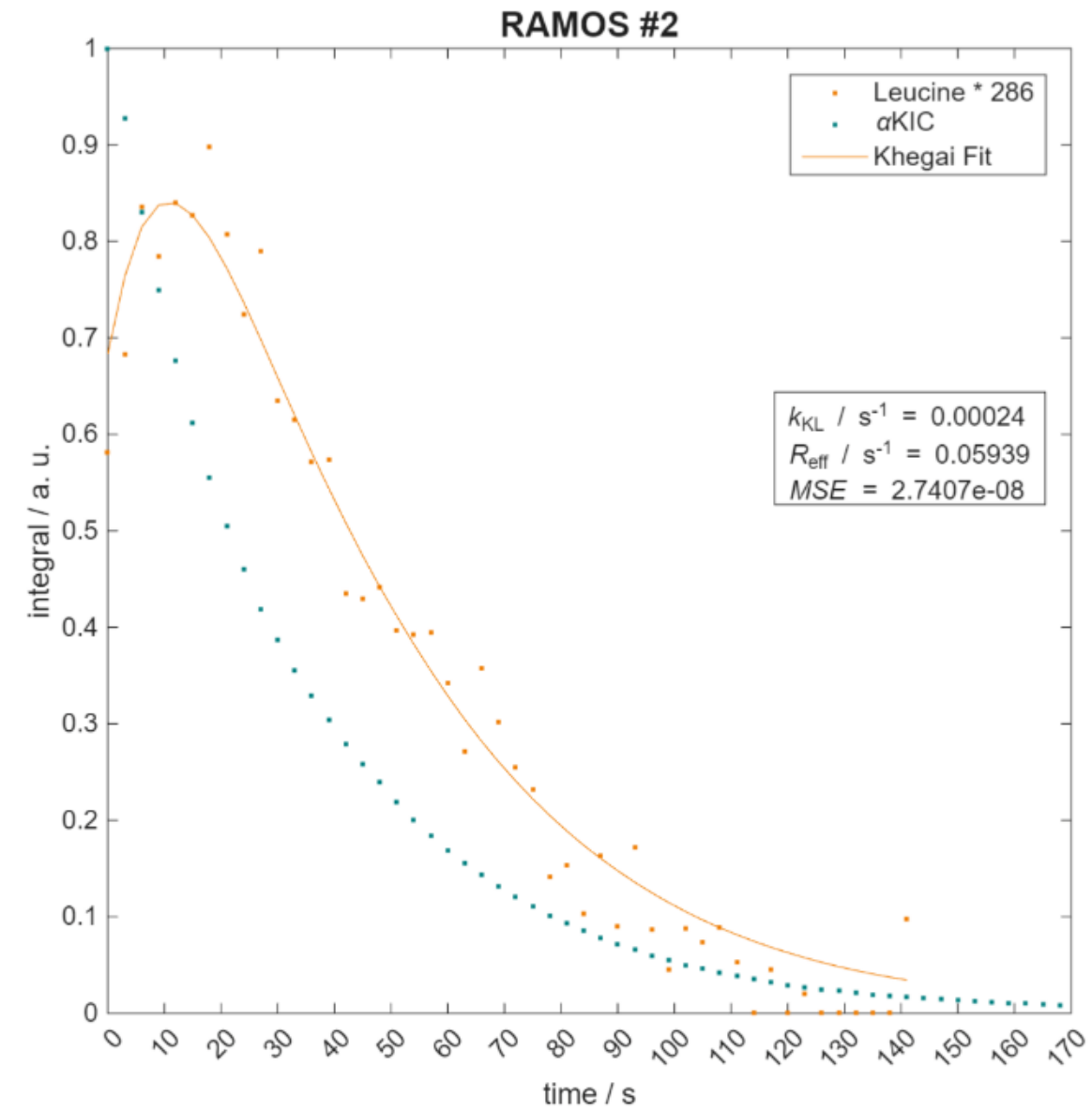

Figure S7 Normalized αKIC (cyan, dots), leucine (orange, dots), and recalculated leucine integrals (orange, line; here Khegai Fit) over time using the RAMOS cell line (*n* = 2). Leucine integrals scaled by a factor of 286 for visibility. Rate constant (k_{KL}), relaxation rate (R_{eff}) and mean-squared error (*MSE*) of the best fit are shown on the right.

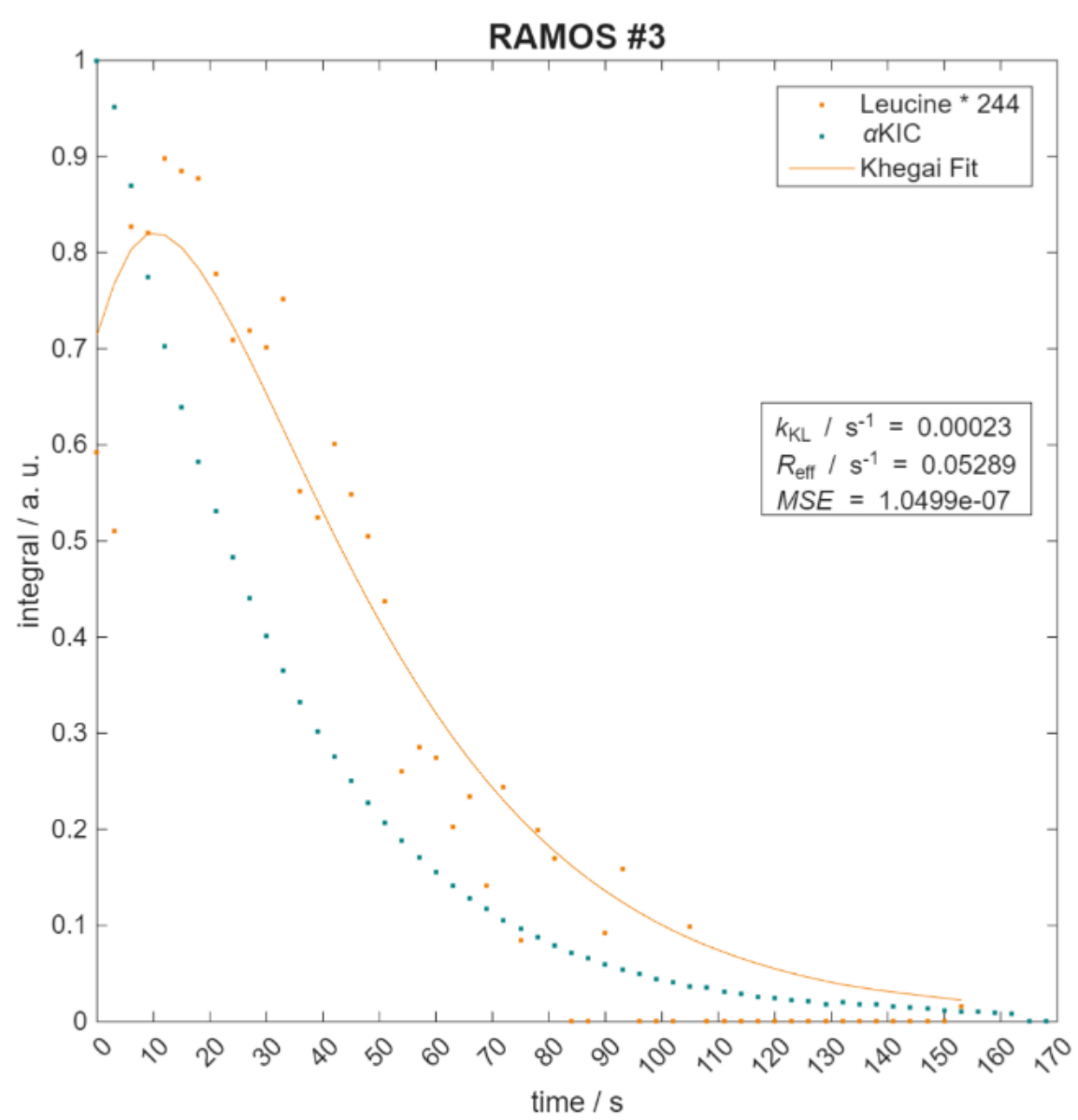

Figure S8 Normalized αKIC (cyan, dots), leucine (orange, dots), and recalculated leucine integrals (orange, line; here Khegai Fit) over time using the RAMOS cell line (*n* = 3). Leucine integrals scaled by a factor of 244 for visibility. Rate constant (k_{KL}), relaxation rate (R_{eff}) and mean-squared error (*MSE*) of the best fit are shown on the right.

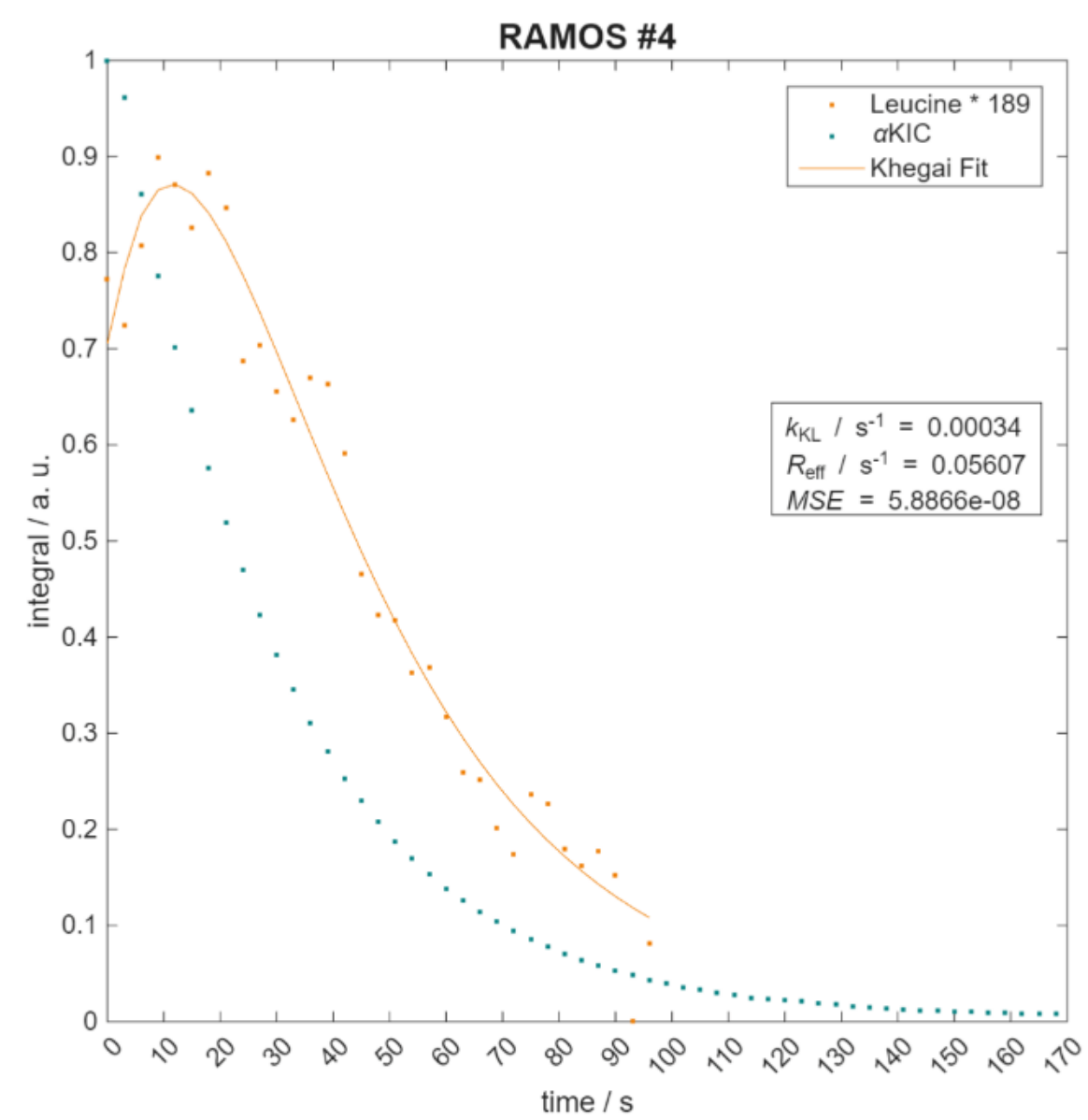

Figure S9 Normalized αKIC (cyan, dots), leucine (orange, dots), and recalculated leucine integrals (orange, line; here Khegai Fit) over time using the RAMOS cell line (*n* = 4). Leucine integrals scaled by a factor of 189 for visibility. Rate constant (k_{KL}), relaxation rate (R_{eff}) and mean-squared error (*MSE*) of the best fit are shown on the right.

DG-75:

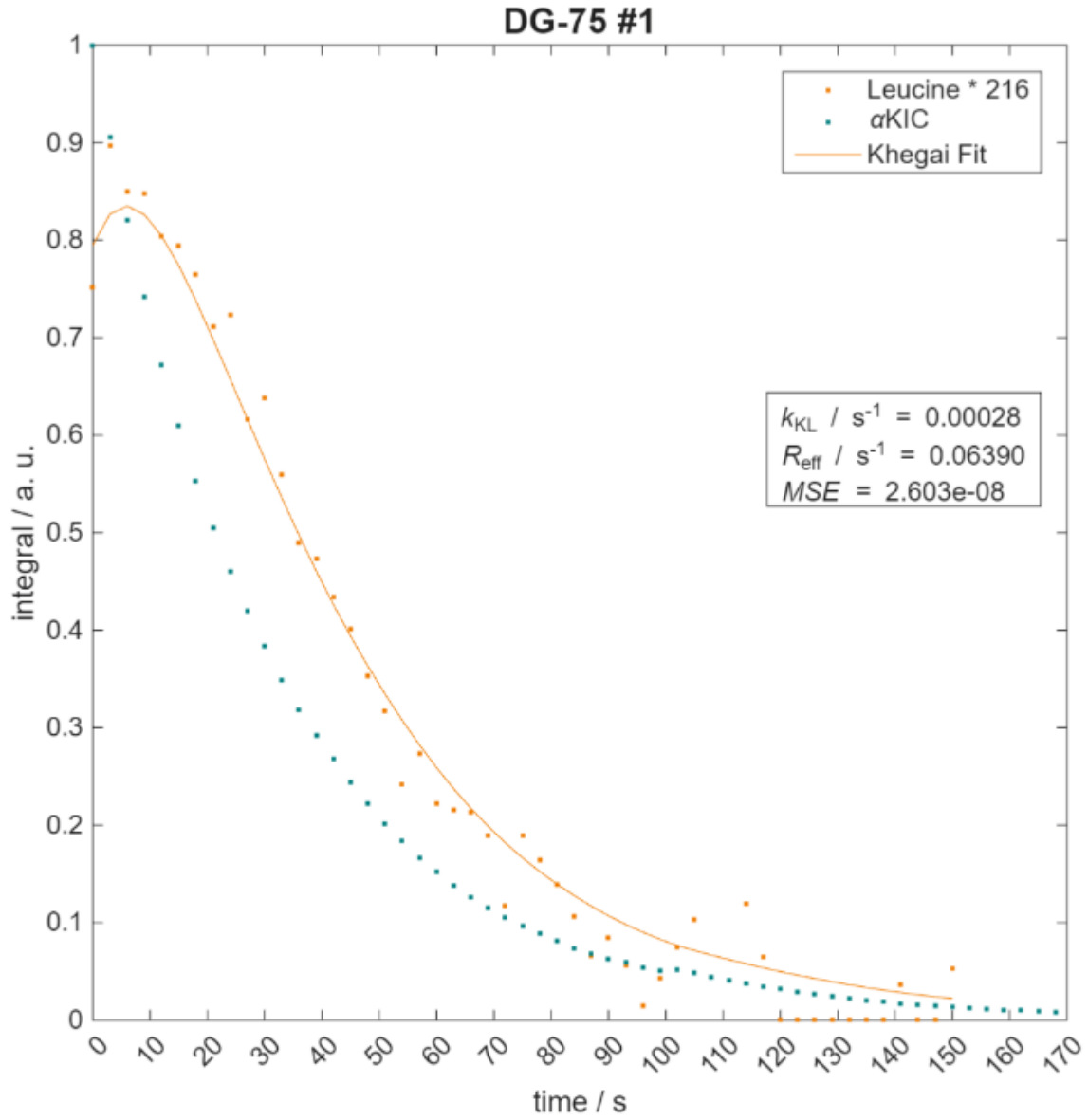

Figure S10 Normalized αKIC (cyan, dots), leucine (orange, dots), and recalculated leucine integrals (orange, line; here Khegai Fit) over time using the RAMOS cell line (*n* = 1). Leucine integrals scaled by a factor of 216 for visibility. Rate constant (k_{KL}), relaxation rate (R_{eff}) and mean-squared error (*MSE*) of the best fit are shown on the right.

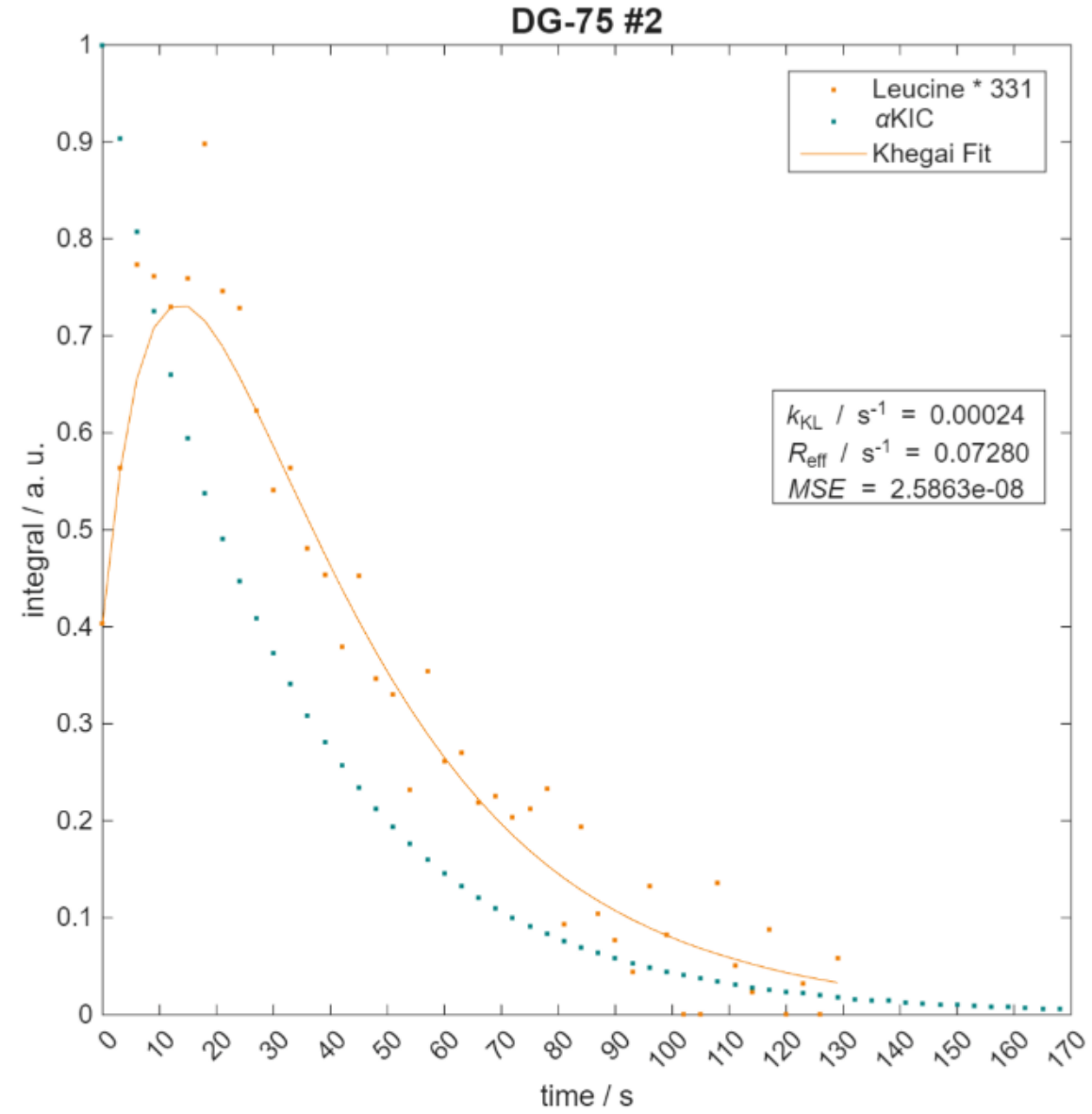

Figure S11 Normalized αKIC (cyan, dots), leucine (orange, dots), and recalculated leucine integrals (orange, line; here Khegai Fit) over time using the RAMOS cell line (*n* = 2). Leucine integrals scaled by a factor of 331 for visibility. Rate constant (k_{KL}), relaxation rate (R_{eff}) and mean-squared error (*MSE*) of the best fit are shown on the right.

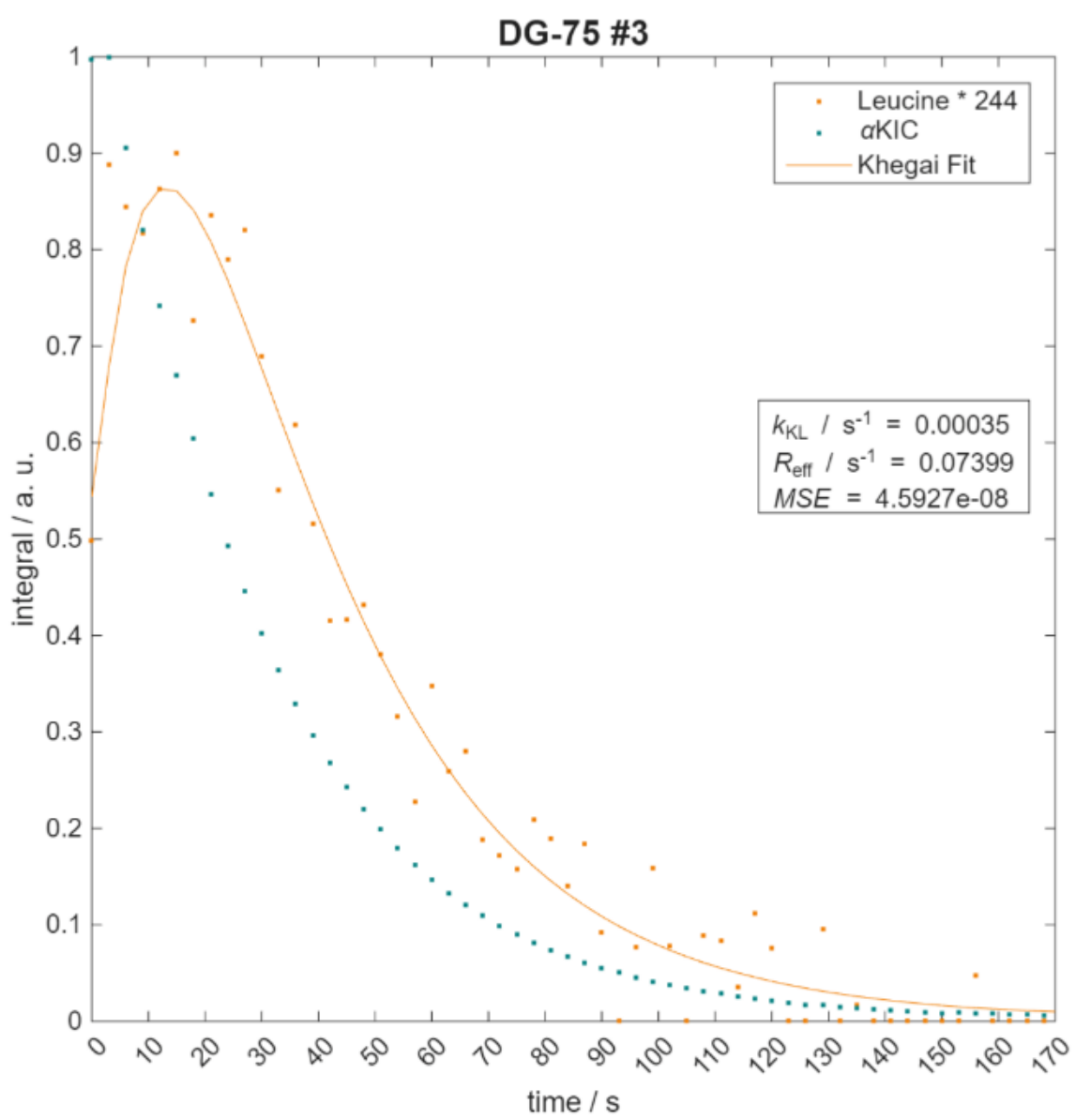

Figure S12 Normalized αKIC (cyan, dots), leucine (orange, dots), and recalculated leucine integrals (orange, line; here Khegai Fit) over time using the RAMOS cell line (*n* = 3). Leucine integrals scaled by a factor of 244 for visibility. Rate constant (k_{KL}), relaxation rate (R_{eff}) and mean-squared error (*MSE*) of the best fit are shown on the right.

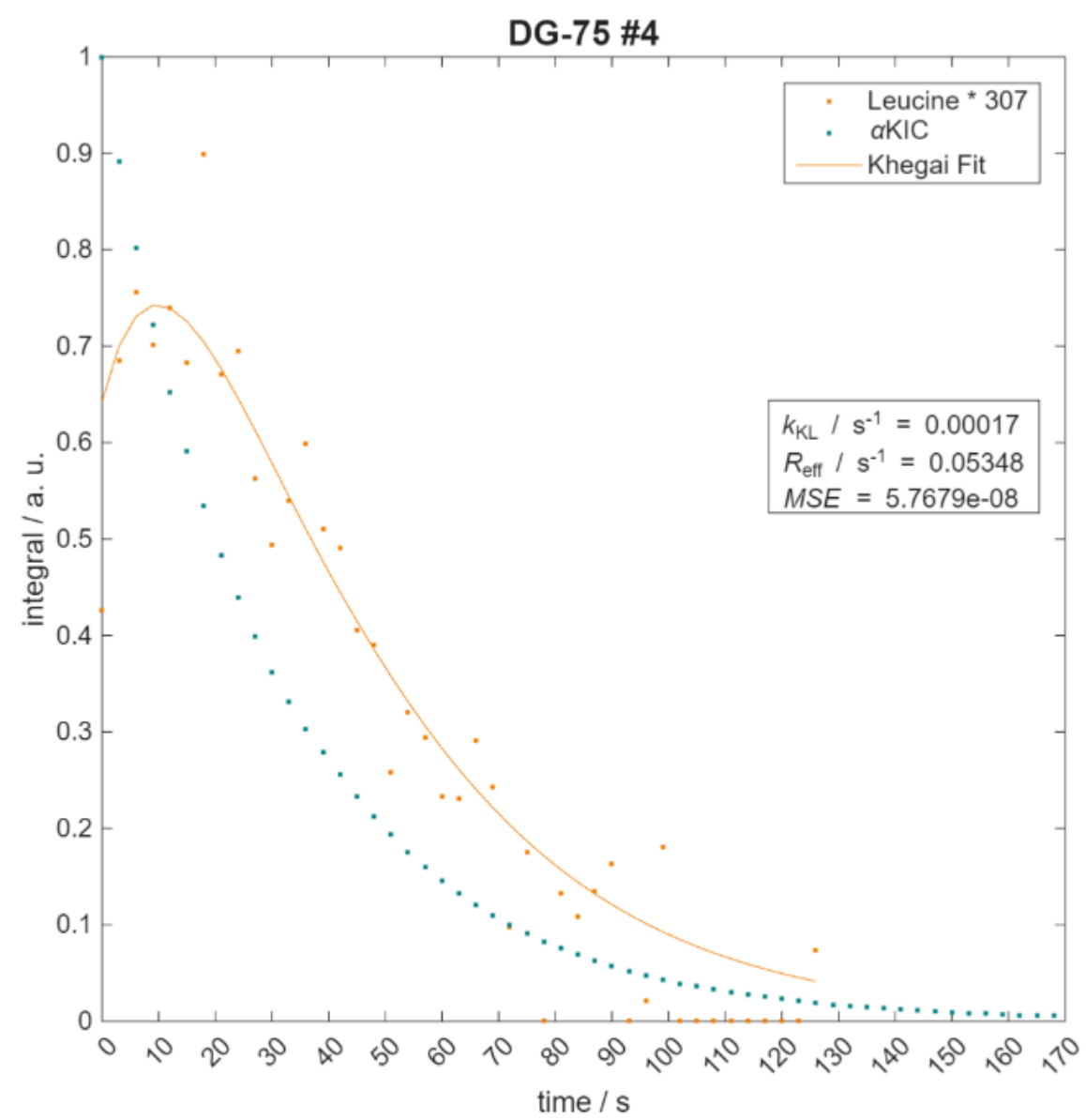

Figure S13 Normalized αKIC (cyan, dots), leucine (orange, dots), and recalculated leucine integrals (orange, line; here Khegai Fit) over time using the RAMOS cell line (*n* = 4). Leucine integrals scaled by a factor of 307 for visibility. Rate constant (k_{KL}), relaxation rate (R_{eff}) and mean-squared error (*MSE*) of the best fit are shown on the right.

JURKAT:

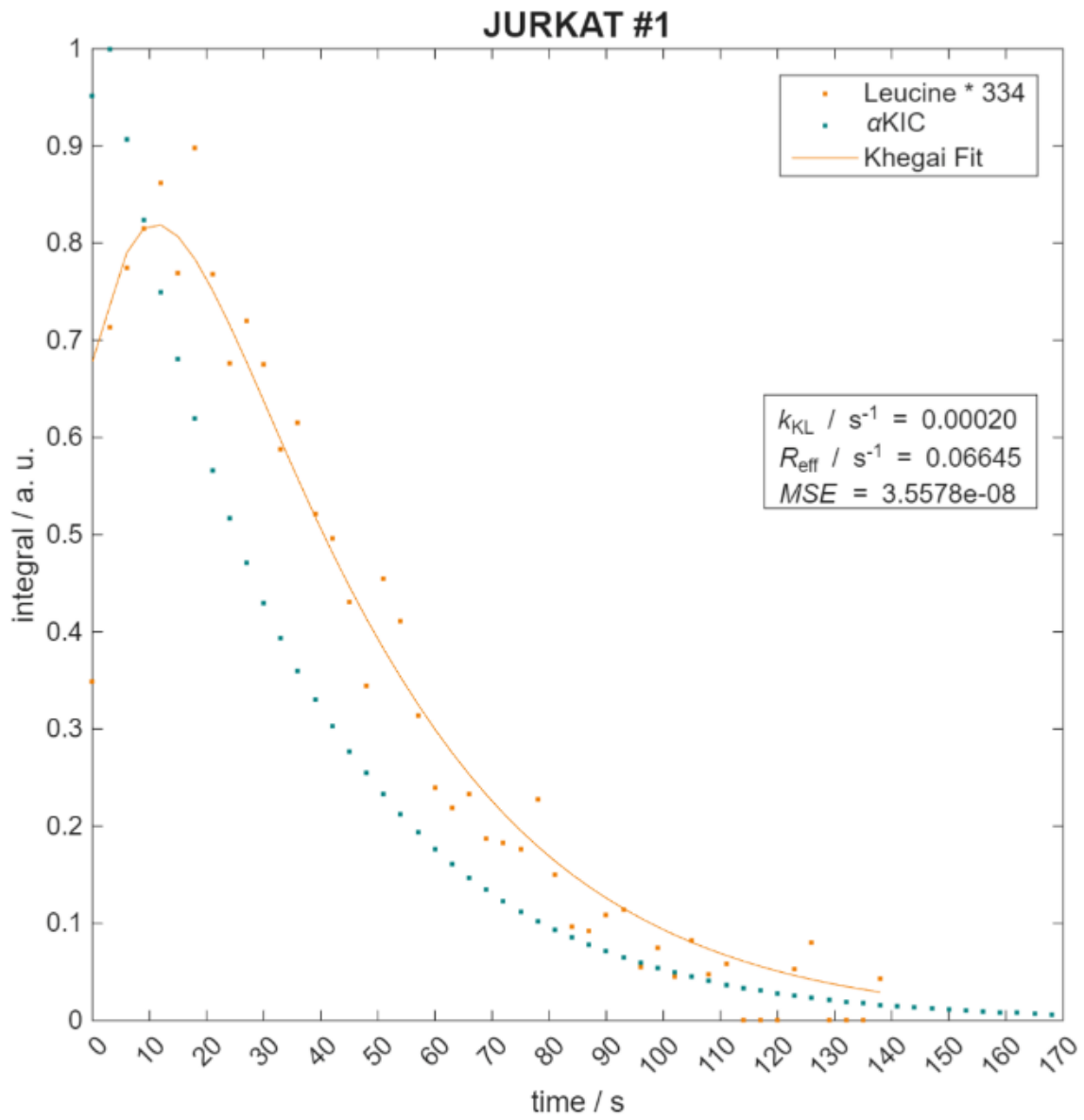

Figure S14 Normalized αKIC (cyan, dots), leucine (orange, dots), and recalculated leucine integrals (orange, line; here Khegai Fit) over time using the RAMOS cell line ($n = 1$). Leucine integrals scaled by a factor of 334 for visibility. Rate constant (k_{KL}), relaxation rate (R_{eff}) and mean-squared error (*MSE*) of the best fit are shown on the right.

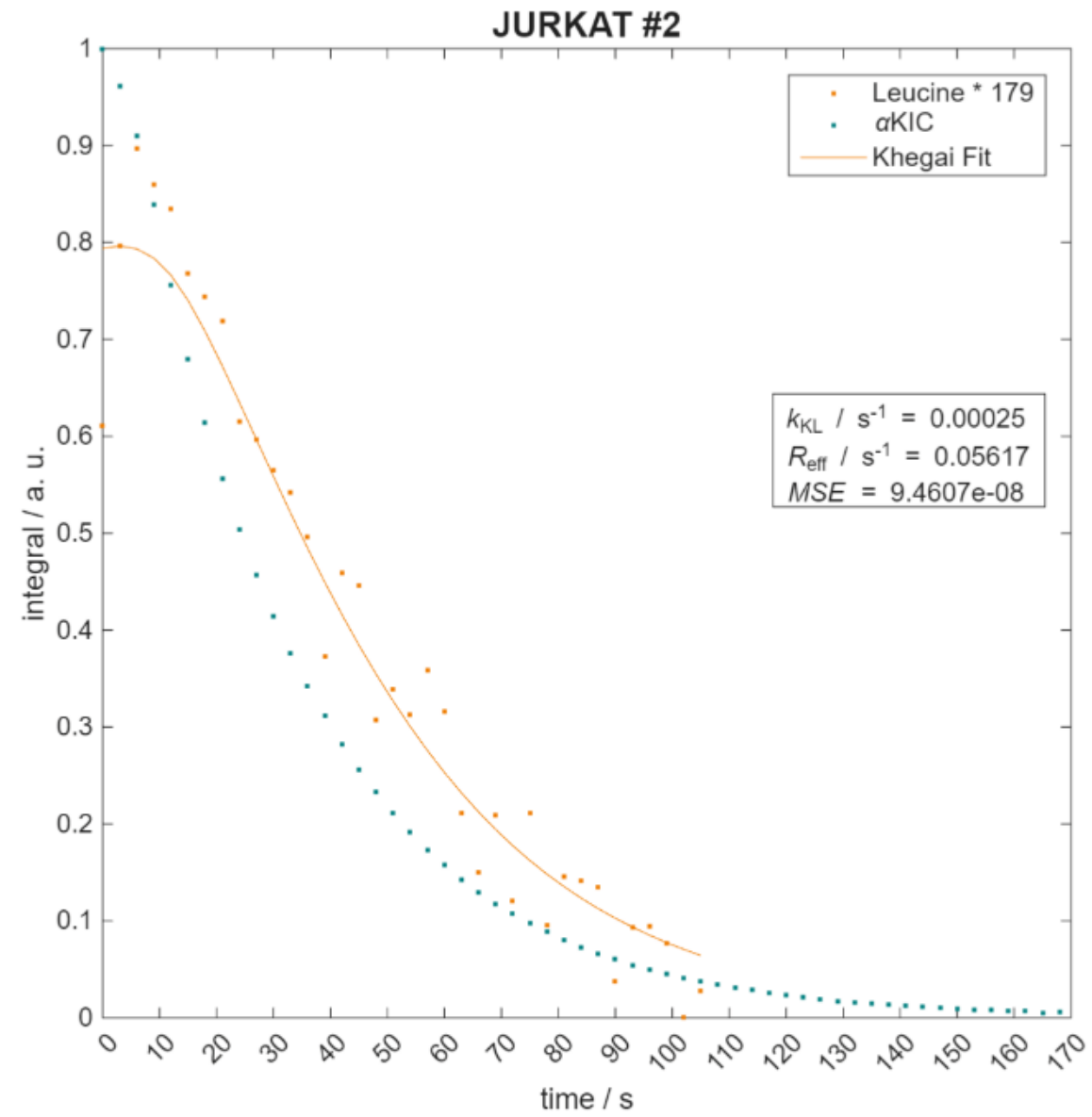

Figure S15 Normalized αKIC (cyan, dots), leucine (orange, dots), and recalculated leucine integrals (orange, line; here Khegai Fit) over time using the RAMOS cell line ($n = 2$). Leucine integrals scaled by a factor of 179 for visibility. Rate constant (k_{KL}), relaxation rate (R_{eff}) and mean-squared error (*MSE*) of the best fit are shown on the right.

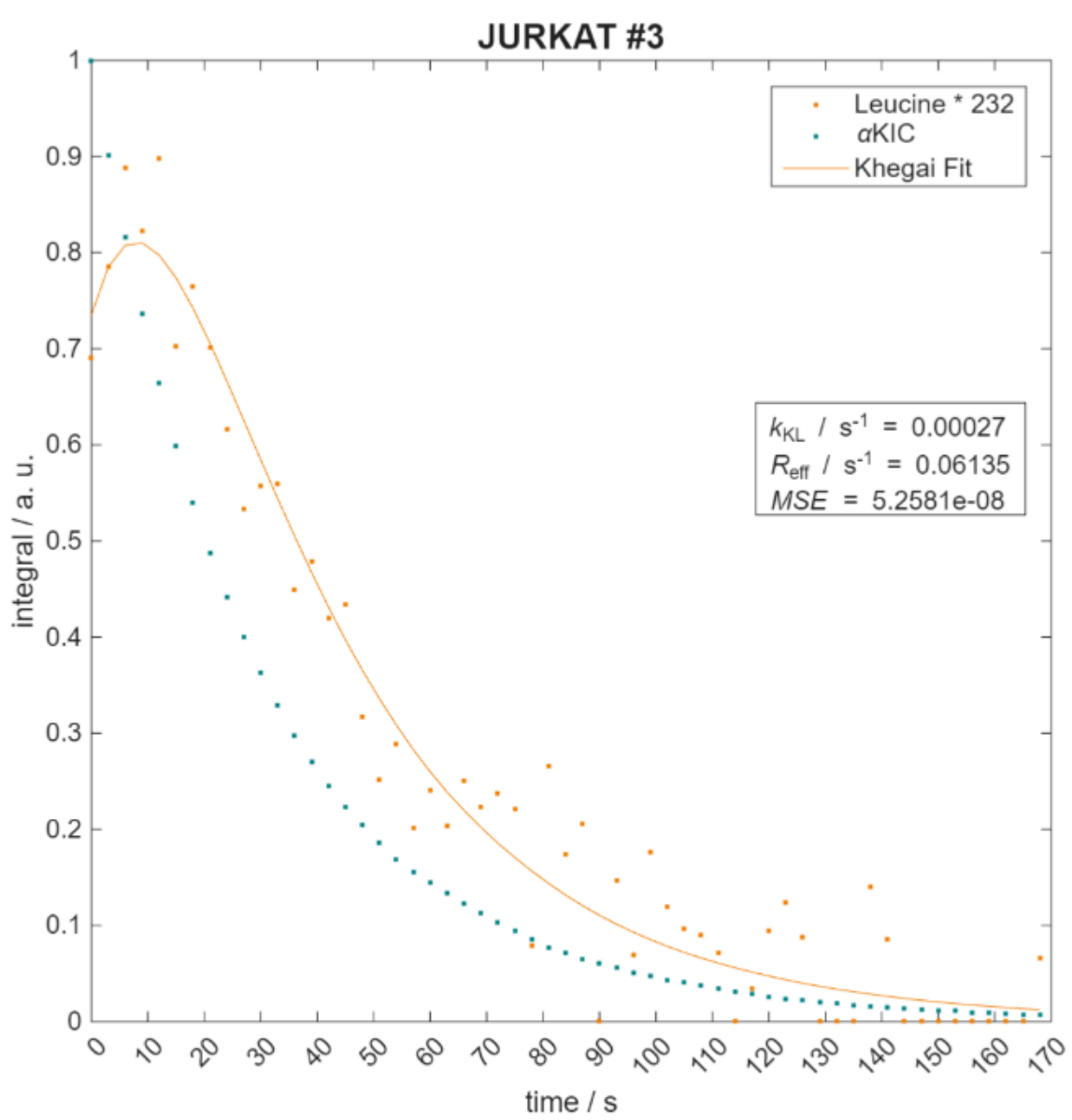

Figure S16 Normalized αKIC (cyan, dots), leucine (orange, dots), and recalculated leucine integrals (orange, line; here Khegai Fit) over time using the RAMOS cell line ($n = 3$). Leucine integrals scaled by a factor of 232 for visibility. Rate constant (k_{KL}), relaxation rate (R_{eff}) and mean-squared error (*MSE*) of the best fit are shown on the right.

Spectra

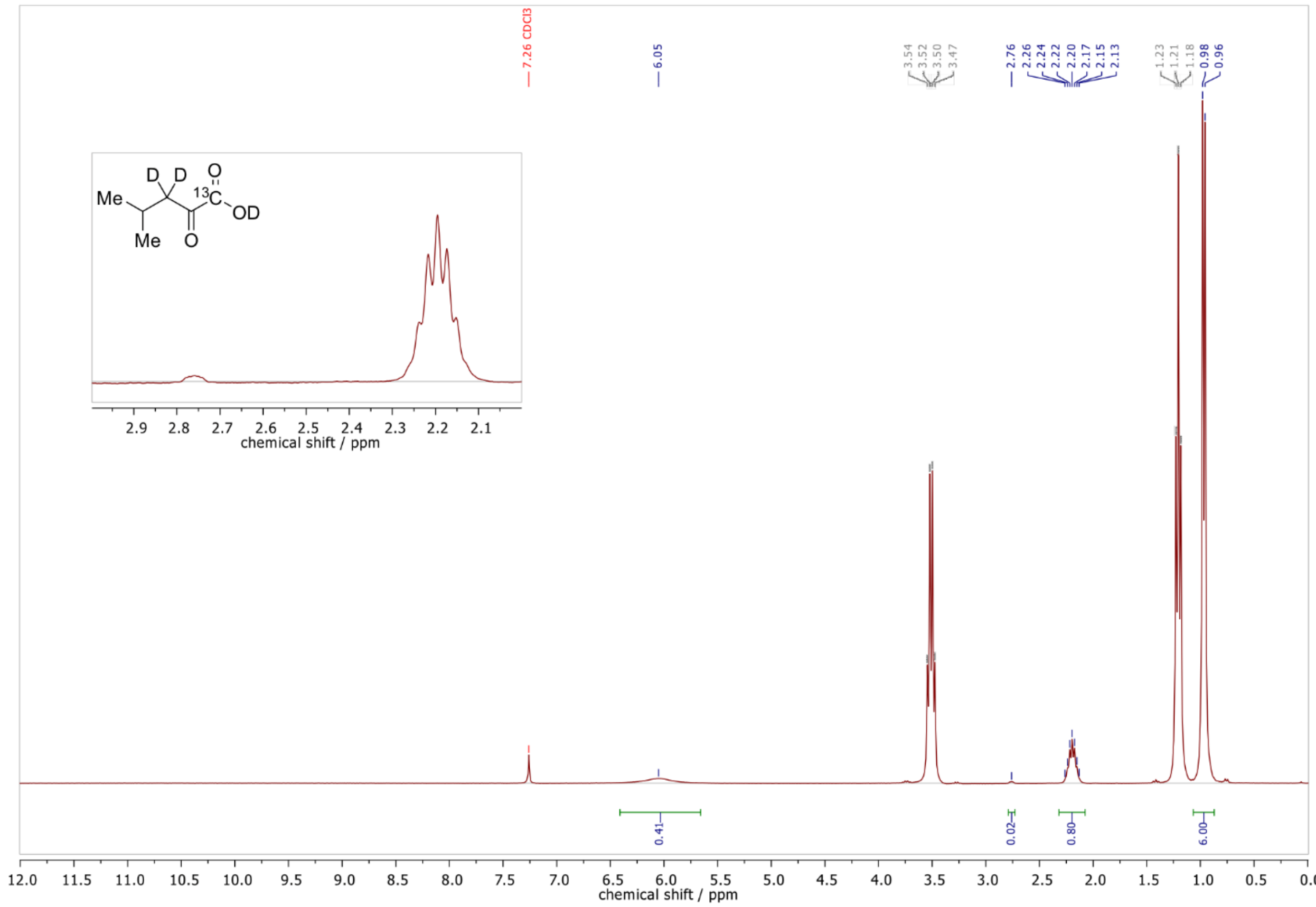

Figure S17 ^{1}H NMR (300 MHz, $CDCl_3$) spectrum of [1-^{13}C]α-ketoisocaproic acid-3,3-d_3.

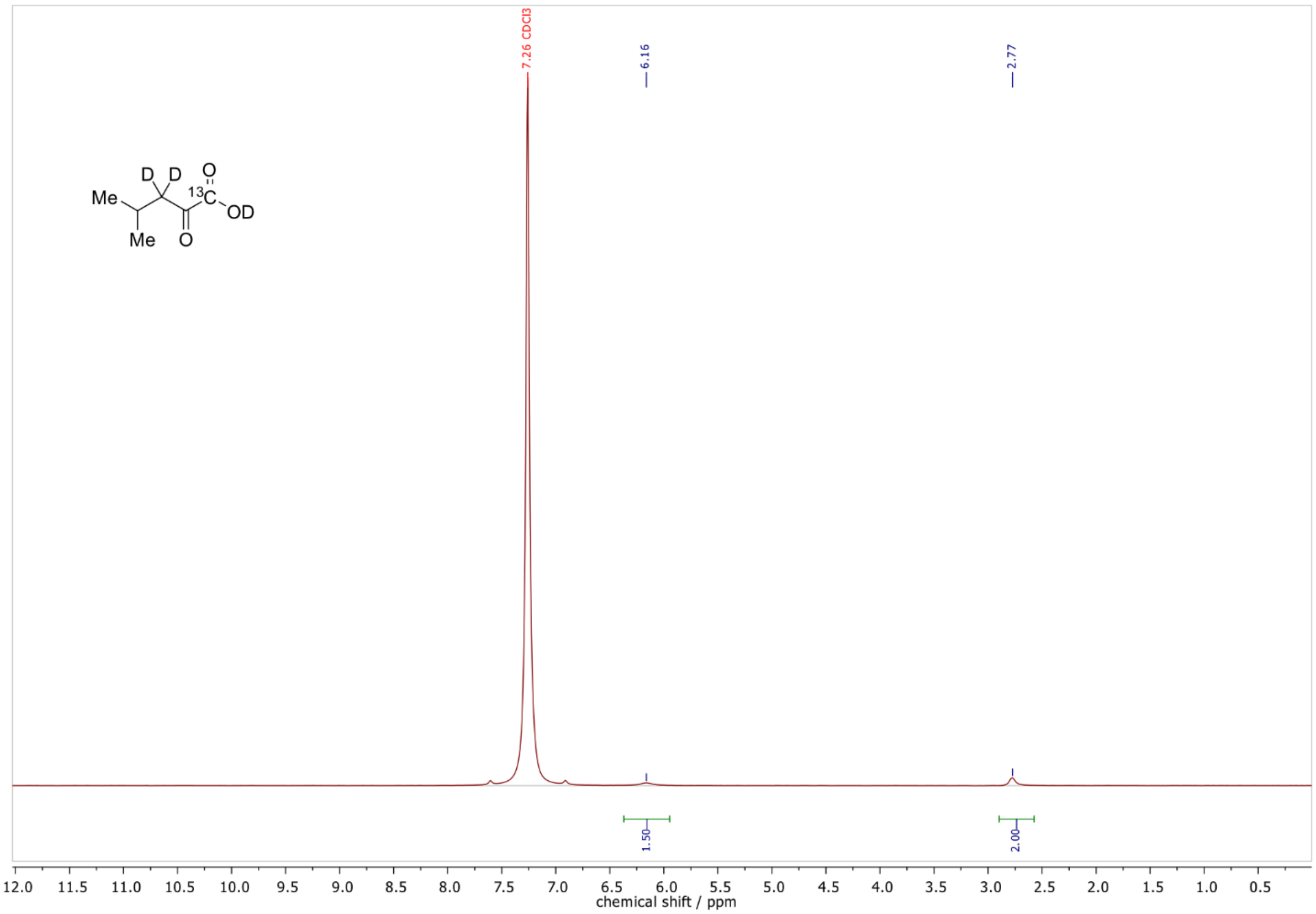

Figure S18 ^{2}H NMR (46 MHz, $CDCl_3$) spectrum of [1-^{13}C]α-ketoisocaproic acid-3,3-d_3.

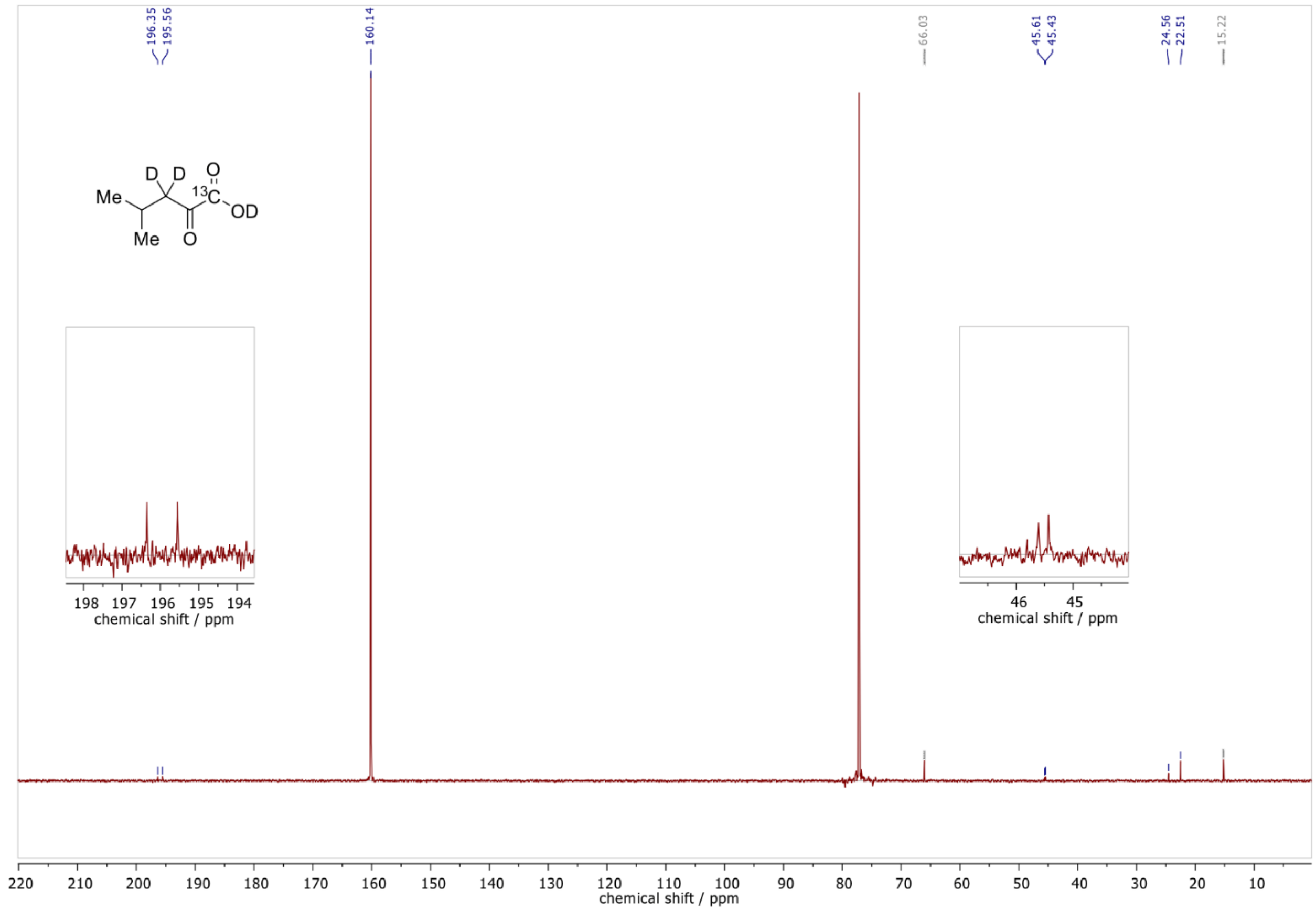

Figure S19 $^{13}C\{^{1}H, ^{2}H\}$ NMR (75 MHz, $CDCl_3$) spectrum of [1-^{13}C]α-ketoisocaproic acid-3,3-*d*$_3$.

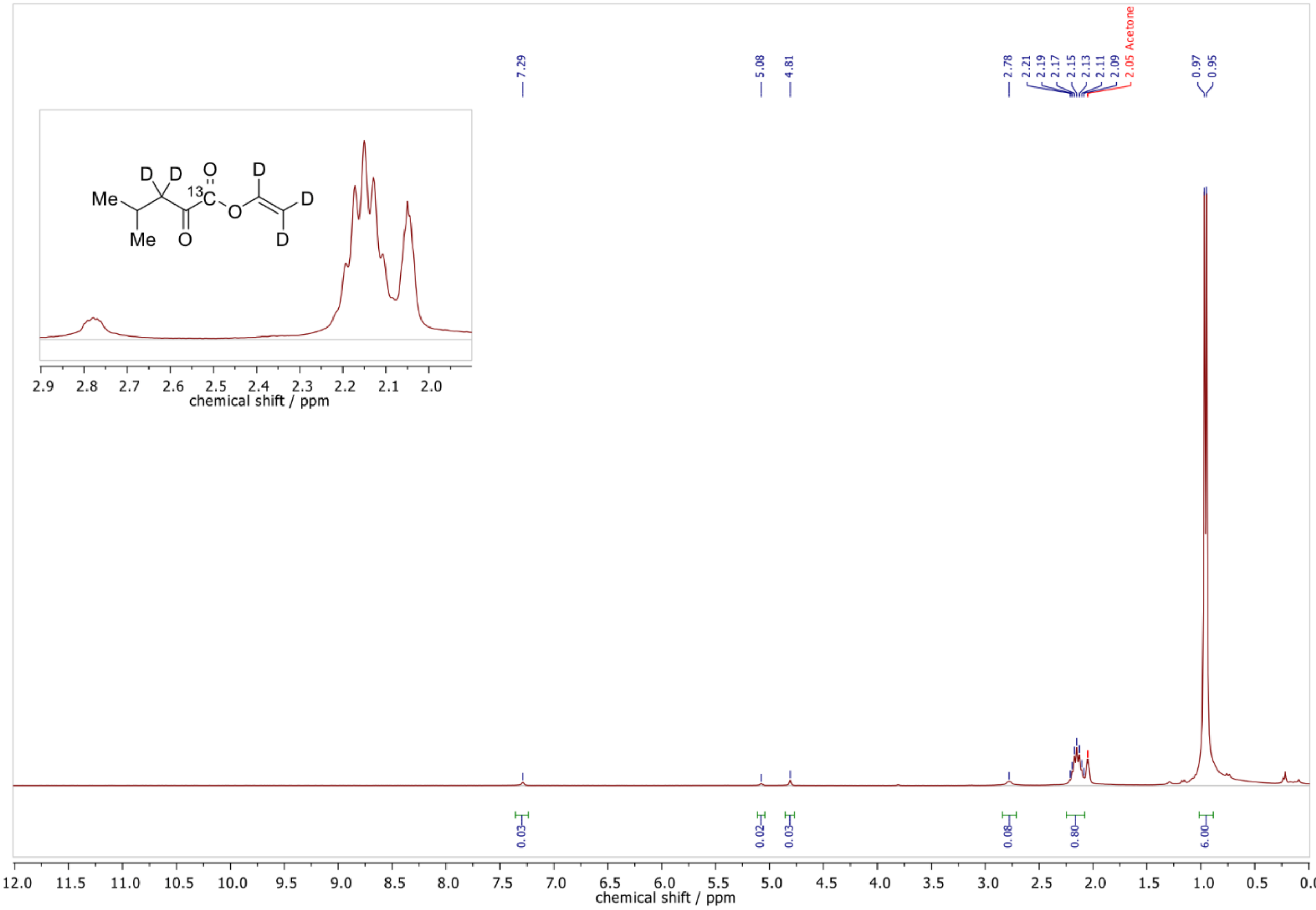

Figure S20 ^{1}H NMR (300 MHz, acetone-d_6) spectrum of vinyl-d_3 [1-^{13}C]α-ketoisocaproic acid-3,3-d_2.

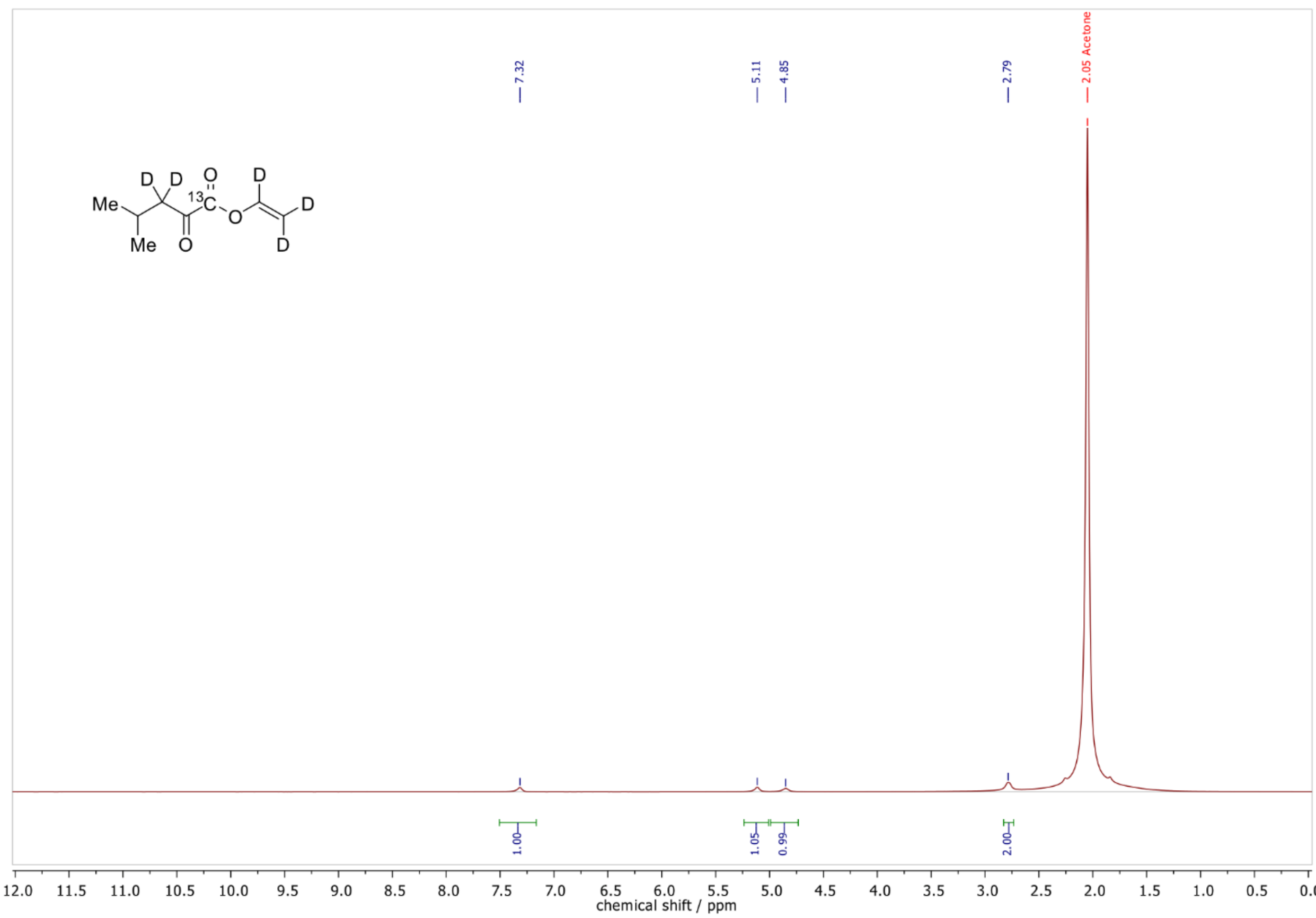

Figure S21 ^{2}H NMR (46 MHz, acetone-d_6) spectrum of vinyl-d_3 [1-^{13}C]α-ketoisocaproic acid-3,3-d_2.

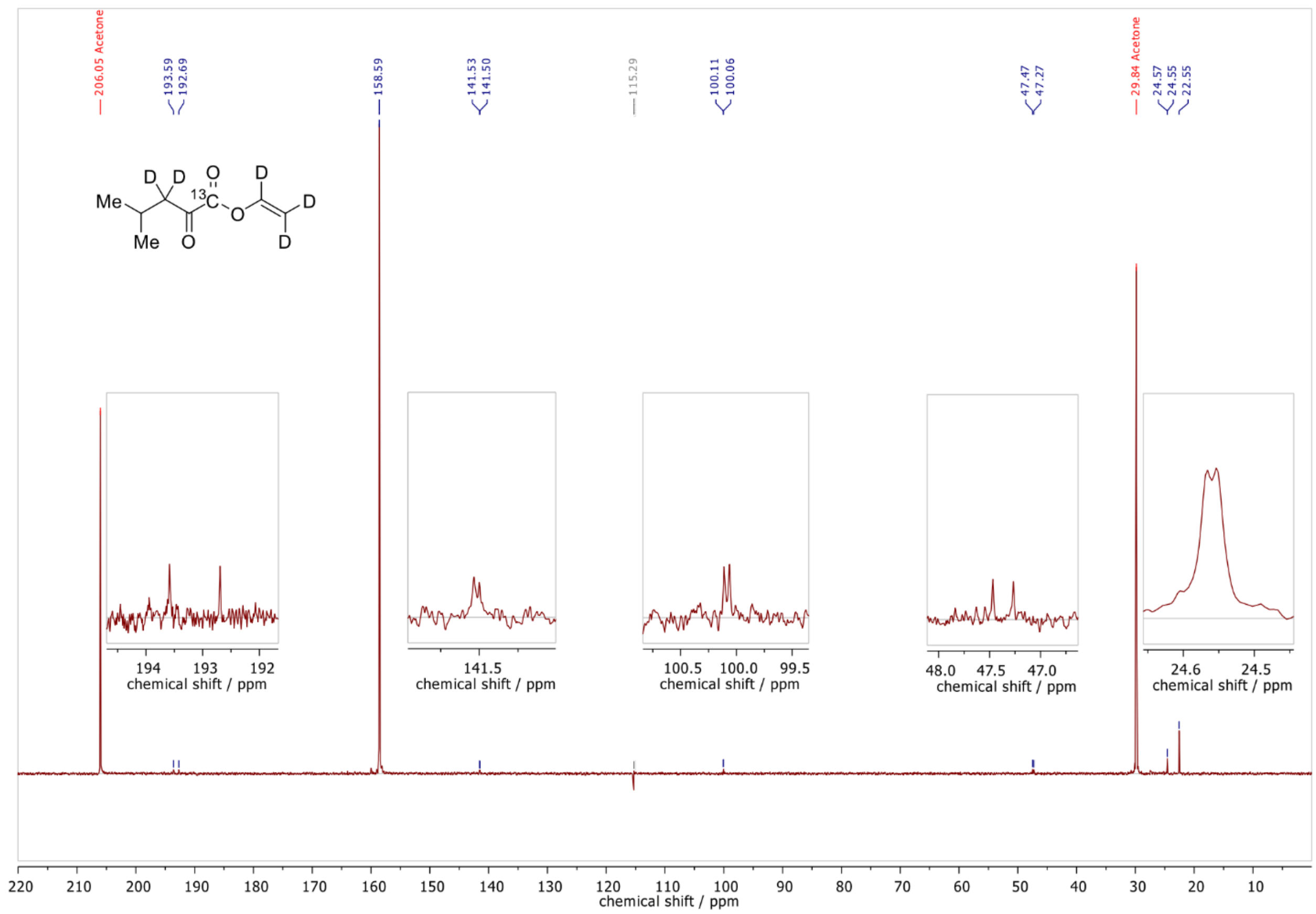

Figure S22 $^{13}C\{^{1}H, ^{2}H\}$ NMR (75 MHz, acetone-d_6) spectrum of vinyl-d_3 [1-^{13}C]α-ketoisocaproic acid-3,3-d_2.

References

(1) *GraphPad Prism, version 10.4.1 (627)*; *GraphPad Prism, version 10.4.1 (627)*, GraphPad Software Inc.: Boston, Massachusetts, 2024.

(2) Hesp, K. D.; Tobisch, S.; Stradiotto, M. $[Ir(COD)Cl]_2$ as a catalyst precursor for the intramolecular hydroamination of unactivated alkenes with primary amines and secondary alkyl- or arylamines: a combined catalytic, mechanistic, and computational investigation. *J. Am. Chem. Soc.* **2010**, *132* (1), 413–426. DOI: 10.1021/ja908316n.

(3) Montgomery, C. L.; Amtawong, J.; Jordan, A. M.; Kurtz, D. A.; Dempsey, J. L. Proton transfer kinetics of transition metal hydride complexes and implications for fuel-forming reactions. *Chem. Soc. Rev.* **2023**, *52* (20), 7137–7169. DOI: 10.1039/d3cs00355h.

(4) Werner, H.; Höhn, A. Synthese und reaktivität des diphenylacetyleniridium-komplexes $C_5H_5Ir(C_2Ph_2)PPr_3^i$ und bildung einer isomeren iridainden-verbindung. *J. Organomet. Chem.* **1984**, *272* (1), 105–113. DOI: 10.1016/0022-328x(84)80446-5.

(5) Werner, H.; Wolf, J.; Alonso, F. J. G.; Ziegler, M. L.; Serhadli, O. Vinylidene transition-metal complexes. *J. Organomet. Chem.* **1987**, *336* (3), 397–411. DOI: 10.1016/0022-328x(87)85200-2.

(6) Höhn, A.; Werner, H. Vinyliden-Übergangsmetallkomplexe. *J. Organomet. Chem.* **1990**, *382* (1-2), 255–272. DOI: 10.1016/0022-328x(90)85231-m.

(7) Wang, Z. X.; Li, B. J. Construction of Acyclic Quaternary Carbon Stereocenters by Catalytic Asymmetric Hydroalkynylation of Unactivated Alkenes. *J. Am. Chem. Soc.* **2019**, *141* (23), 9312–9320. DOI: 10.1021/jacs.9b03027.

(8) Esteruelas, M. A.; García, M. P.; Martín, M.; Nürnberg, O.; Oro, L. A.; Werner, H. Reactions of $[IrH_2(Me_2CO)(Hpz)(PPh_3)_2]BF_4$ with alkynes: synthesis of new hydride-vinyl iridium(III) complexes. *J. Organomet. Chem.* **1994**, *466* (1-2), 249–257. DOI: 10.1016/0022-328x(94)88052-2.

(9) Atencio, R.; Bohanna, C.; Esteruelas, M. A.; Lahoz, F. J.; Oro, L. A. Synthesis, reactivity and catalytic activity of $[RuH(\eta^1\text{-}OCMe_2)(CO)_2(PPr^{i3})_2]BF_4$. *J. Chem. Soc., Dalton Trans.* **1995**, (13), 2171–2181. DOI: 10.1039/dt9950002171.

(10) Echavarren, A. M.; Lopez, J.; Santos, A.; Romero, A.; Hermoso, J. A.; Vegas, A. Reactions of cationic ruthenium hydrides with 1-alkynes: formation of *sigma*-alkynyl ruthenium complexes and reduction of 1-alkynes to 1-alkenes. *Organometallics* **1991**, *10* (7), 2371–2376. DOI: 10.1021/om00053a046.

(11) Werner, H.; Meyer, U.; Peters, K.; von Schnering, H. G. Fünf- und sechsfach koordinierte Vinylruthenium- und -osmium-Komplexe durch Alkin-Insertion in Hydridometall-Vorstufen. *Chem. Ber.* **1989**, *122* (11), 2097–2107. DOI: 10.1002/cber.19891221109.

(12) Esteruelas, M. A.; Lahoz, F. J.; Lopez, J. A.; Oro, L. A.; Schluenken, C.; Valero, C.; Werner, H. Synthesis, molecular structure, and reactivity of octahedral alkylhydridoosmium(II) complexes $[OsH(R)(CO)_2(PR'_3)_2]$. *Organometallics* **1992**, *11* (6), 2034–2043. DOI: 10.1021/om00042a018.

(13) Van der Zeijden, A. A. H.; Bosch, H. W.; Berke, H. Insertion of acetylenes into tungsten hydride complex trans,trans-$WH(CO)_2(NO)(PMe_3)_2$. *Organometallics* **1992**, *11* (2), 563–573. DOI: 10.1021/om00038a011.

(14) *TopSpin, version 4.5.0*; *TopSpin, version 4.5.0*, Bruker Corporation: Billerica, Massachusetts, 2019.

(15) *Matlab R2025a*; *MATLAB, version 25.1.0.2943329 (R2025a)*, The MathWorks Inc.: Natick, Massachusetts, 2025.

(16) Khegai, O.; Schulte, R. F.; Janich, M. A.; Menzel, M. I.; Farrell, E.; Otto, A. M.; Ardenkjaer-Larsen, J. H.; Glaser, S. J.; Haase, A.; Schwaiger, M.; et al. Apparent rate constant mapping using hyperpolarized [1-^{13}C]pyruvate. *NMR Biomed.* **2014**, *27* (10), 1256–1265. DOI: 10.1002/nbm.3174.

(17) Jin, H.; Zhang, C.; Zwahlen, M.; von Feilitzen, K.; Karlsson, M.; Shi, M.; Yuan, M.; Song, X.; Li, X.; Yang, H.; et al. Systematic transcriptional analysis of human cell lines for gene expression landscape and tumor representation. *Nat. Commun.* **2023**, *14* (1), 5417. DOI: 10.1038/s41467-023-41132-w.

(18) The Human Protein Atlas, "proteinatlas.org", can be found under https://proteinatlas.org/, **2026** (accessed: 03.03.2026).
